# Triplet Energy Transfer Strategy for Visible Light Activation of Bio‐Based Oxime Esters in Photopolymerization and 3D Printing

**DOI:** 10.1002/smll.74715

**Published:** 2026-07-23

**Authors:** Kaidan Yang, Céline Dietlin, Coralie Ohl, Margot Manivel, Fabrice Morlet‐Savary, Michael Schmitt, Gérard Audran, Jean‐Patrick Joly, Jing Zhang, Pu Xiao, Jacques Lalevée

**Affiliations:** ^1^ CNRS IS2M UMR7361 Université De Haute‐Alsace Mulhouse France; ^2^ Université De Strasbourg Strasbourg France; ^3^ CNRS ICR UMR7273 Aix Marseille Université Marseille France; ^4^ Future Industries Institute Adelaide University Mawson Lakes South Australia Australia; ^5^ School of Chemical Engineering Adelaide University Adelaide Australia; ^6^ State Key Laboratory of High Performance Ceramics Shanghai Institute of Ceramics Chinese Academy of Sciences Shanghai People's Republic of China; ^7^ Institut Universitaire de France Paris France

**Keywords:** 3D printing, bio‐based oxime ester, dexter‑type triplet–triplet energy transfer, visible light/sunlight photopolymerization

## Abstract

Visible‐light and solar photopolymerization require photoinitiating systems that combine high reactivity, formulation stability, and biological safety. Here, we present a triplet energy transfer strategy that couples bio‐based benzoyl oxime esters, prepared from bio‐derived aldehydes, with the high‐triplet‐energy visible sensitizer 2‐isopropylthioxanthone (ITX), converting UV‐absorbing oxime ester scaffolds into efficient metal‐free photoinitiators for 405 nm LED and sunlight irradiation. Quantum chemical calculations, electrochemical analysis, laser flash photolysis, ESR spin trapping, and in situ FTIR reveal that Dexter‐type triplet–triplet energy transfer from ITX selectively populates the reactive triplet states of the oxime esters, while the electron pathway is disfavored. Subsequent N─O bond cleavage and rapid decarboxylation generate initiating phenyl radicals. The BE2+ITX formulation affords faster curing, higher conversion, and deeper through‐cure than BE2 or ITX alone, while matching or surpassing benchmark onium and amine systems with favorable cytocompatibility and storage stability. This triplet‐energy‐driven design rule enables modular development of greener photoinitiators for high‐resolution 3D printing and ambient curing.

## Graphical abstract

Bio‐based oxime esters are converted into efficient metal‐free photoinitiators for 405 nm LEDs and sunlight by Dexter‑type triplet energy transfer from ITX, enabling fast and deep visible light curing, high‐resolution 3D printing and improved cytocompatibility.

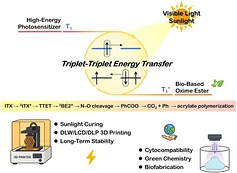

## 1 Introduction

Photoinitiated radical polymerization underpins a broad range of technologies, from protective coatings, adhesives, and microelectronics to dental composites and additive manufacturing [1]. Over the past decade, there has been a strong shift from mercury lamps and near‐UV sources toward compact visible light LEDs and even direct use of sunlight [2]. This shift is driven by the need for safer curing conditions, lower energy consumption, and better compatibility with colored or filled formulations, as well as by the rapid growth of 3D printing and bio‐fabrication platforms that integrate light‐based curing with digital design [3]. Visible light penetrates more deeply into many resins (potentially filled) and is less damaging to cells and tissues, which makes visible light photopolymerization an attractive basis for next‐generation high‐resolution manufacturing and biomedical devices [4]. Realizing this potential, however, depends critically on the availability of photoinitiating systems that can efficiently harvest visible light and solar photons while meeting stringent requirements on reactivity, stability, and biological safety.

Recent studies have introduced a variety of visible light photoinitiators and multi‐component photoinitiating systems that extend the usable wavelength range from the blue into the green, [5] red, and even near‐infrared regions [6]. Metal‐based complexes, organic dyes, and type II combinations with amine or onium co‐initiators can generate radicals under visible light irradiation and have enabled important advances in 3D printing and thick sample curing [7]. Despite this progress, trade‐offs remain between initiation efficiency, formulation stability, cost, and toxicity [8]. Many highly reactive systems rely on heavy metals for visible‐light photoredox polymerizations [9] or strongly colored chromophores that are not ideal for large‐scale or biomedical use [10]. Others suffer from limited penetration depth, oxygen sensitivity, or long‐term instability in storage, which complicates process control in industrial environments [11]. Design strategies that tune the absorption profile of a photoinitiator through extended conjugation can improve visible light harvesting, but they often increase synthetic complexity and can compromise solubility or compatibility with polar or bio‐based monomers [12]. There is therefore considerable interest in approaches that retain the simple, modular structures of established radical precursors while enabling their activation under visible light.

Oxime esters have emerged as a versatile class of radical photoinitiators because their excited states undergo homolytic N─O bond cleavage followed by rapid decarboxylation to generate highly reactive carbon‐centered radicals [13]. In addition, oxime ester scaffolds can be synthesized in only a few steps from commercially available or bio‐derived aldehydes, which makes them attractive building blocks for more sustainable photoinitiating systems [14]. However, most simple oxime esters absorb predominantly in the near UV region and are essentially transparent above about 350 nm, often undergoing Type I cleavage under UV light to generate free radicals [15]. This makes them inefficient at harvesting visible light and restricts their direct use in modern visible light LED‐driven processes. To extend their absorption in the visible range, more complex oxime esters must be synthesized on extended chromophores, which complicates their synthesis pathways and can lead to storage stability issues. This intrinsic limitation has, in turn, motivated growing efforts to develop sensitization strategies that relay visible light excitation from suitable photosensitizers to simple oxime esters and thereby unlock their reactivity under mild irradiation conditions. Recent near‐infrared sensitization studies have combined strongly absorbing cyanine dyes with oxime esters so that a thermally assisted photoinduced electron transfer from the excited dye to the oxime ester triggers N─O bond cleavage and decarboxylation, thereby generating initiating radicals even under 805–870 nm irradiation [16]. In this work, we instead focus on simple oxime esters directly accessible from bio‐based aldehydes and activate their N─O cleavage under visible light via sensitization with a well‐established photosensitizer, thereby providing a robust and broadly applicable approach.

Energy transfer sensitization offers a conceptually simple route to reconcile this mismatch by decoupling light absorption from radical generation. In many areas of photochemistry and optoelectronics, Förster resonance energy transfer (FRET) between singlet states and Dexter‑type triplet–triplet energy transfer (TTET) have been exploited to funnel excitation energy from a light‐harvesting donor to a reactive acceptor [17]. In small molecule synthesis, visible light photocatalysis has taken this concept further for oxime esters, where triplet energy transfer from appropriately tuned Ir(III) complexes or organic sensitizers populates the triplet states of oxime esters and triggers N─O bond homolysis and decarboxylation, generating acyloxy, alkyl and iminyl radicals that drive highly selective transformations such as decarboxylative C(sp^3^)–N radical–radical cross coupling, [18] vicinal imino–trifluoromethylation of alkenes, [19] and aminative olefin metathesis cascades [20]. In photopolymerization, singlet–singlet FRET pairs that couple optical brighteners with type I acyl phosphine oxide initiators have been used to redistribute excitation within the UV region and significantly enhance curing rates, which illustrates how energy transfer can be harnessed to boost radical generation without changing the excitation wavelength or overall formulation [21]. Mechanistic studies that combine electrochemistry, steady‐state and time‐resolved spectroscopy and DFT calculations have begun to clearly delineate Dexter‑type triplet–triplet energy transfer from competing single‐electron transfer and to emphasize the importance of matching the triplet energy of the photosensitizer to that of the oxime ester while keeping excited‐state redox processes thermodynamically disfavored.

Translating such strategies to photopolymerization is appealing because a visible light photosensitizer can be selected to match the emission of a commercial LED or the solar spectrum, while the chemical structure of the radical precursor can be optimized independently for efficient bond cleavage, low toxicity, and favorable formulation properties. Yet in most reported photoinitiating systems that combine a visible light sensitizer with an UV absorbing initiator, the underlying mechanism is not rigorously established. The relative contributions of singlet–singlet Förster transfer, Dexter triplet exchange and photoinduced electron transfer are often inferred from overall reactivity rather than from comprehensive thermodynamic and kinetic analysis. For oxime ester‐based systems in particular, existing energy transfer studies are largely confined to small molecule functionalization and cross coupling, and there is no general design rule that links triplet energies, redox potentials, and spectral overlap to the efficiency of visible light activation in polymerization settings. As a result, it remains unclear how to systematically choose donor–acceptor pairs that favor triplet energy transfer over competing electron or hydrogen atom transfer processes when the goal is to initiate fast, deep, and spatially controlled curing in practical photopolymer formulations.

Here we introduce an energy transfer sensitization strategy that activates UV‐absorbing benzoyl oxime esters (BEs) under visible light and at the same time provides a broadly applicable design principle for oxime ester‐based photoinitiators prepared from bio‐abundant aldehydes. Using three BEs derived from furfural, vanillin, and syringaldehyde together with several photosensitizers, we combine density functional theory, electrochemistry, and steady‐state spectroscopy to construct the energetic landscape that governs excited‐state cleavage, energy transfer, and electron transfer. Laser flash photolysis, fluorescence quenching, and steady‐state photolysis demonstrates that 2‐isopropylthioxanthone (ITX) can transfer its triplet energy to the BEs with near diffusion‐controlled kinetics, while cyclic voltammetry and frontier orbital analysis rule out thermodynamically favorable photoinduced electron transfer. Electron spin resonance, spin trapping, and in situ infrared spectroscopy further show that triplet sensitization of the oxime esters generates benzoyloxyl radicals, which rapidly decarboxylate with concurrent carbon dioxide evolution to yield phenyl radicals that initiate acrylate polymerization. Under 405 nm LED and under direct sunlight, BE2+ITX formulations exhibit much higher polymerization rates, higher final conversions, and significantly deeper through‐cure than formulations with the sensitizer alone, and they match or slightly surpass benchmark two‐component systems based on onium or amine co‐initiators (e.g., Ethyl 4‐(dimethylamino)benzoate(EDB)+ITX and iodonium salt (Iod)+ITX). The ITX+BE2 two‐component formulation performs robustly across DLW, LCD, and DLP 3D printers and maintains its performance after one month of dark storage, which highlights its practical suitability for high‐resolution 3D printing. Finally, live cell imaging of C_2_C_12_ myoblasts shows that the bio‐based oxime ester‐based system displays favorable short‐term cytocompatibility compared with a conventional amine co‐initiated formulation, underscoring the relevance of this strategy for light‐mediated material formation and future bio‐fabrication. Together, these results establish Dexter‑type triplet–triplet energy transfer from visible light sensitizers to oxime esters as an efficient route to visible light photopolymerization, and they define a simple triplet energy‐driven design rule that can guide the development of greener and more sustainable photoinitiating systems.

## 2 Experimental Section

Detailed experimental procedures, including materials, synthesis and characterization of the bio‐based benzoyl oxime esters, photophysical measurements, electrochemical analysis, photolysis experiments, ESR spin‐trapping, laser flash photolysis, real‐time FTIR photopolymerization, 3D printing tests, computational methods, and cell confluence assays, are provided in the Supporting Information. Briefly, the benzoyl oxime esters were prepared from bio‐derived aldehydes through oxime formation followed by benzoylation. For phenol‐containing substrates, protection and deprotection steps were used to afford the final oxime ester structures. The compounds were characterized by NMR spectroscopy and high‐resolution mass spectrometry.

Photophysical and mechanistic investigations were conducted in acetonitrile or tert‐butylbenzene using UV–vis absorption, fluorescence spectroscopy, cyclic voltammetry, steady‐state photolysis, ESR spin trapping, and laser flash photolysis. Photopolymerization experiments were performed mainly in TMPTA or ETPTA under LED or sunlight irradiation and monitored by real‐time FTIR spectroscopy. Carbon dioxide evolution was followed by the FTIR band at 2337 cm^−1^. The resulting formulations were further evaluated by Jacobs' working curve analysis and 3D printing using DLW, LCD, and DLP platforms. Preliminary cytocompatibility was assessed using C_2_C_12_ myoblast confluence assays. Additional experimental details and data‐processing procedures are given in the Supporting Information.

## 3 Results and Discussion

### 3.1 Synthesis of BEs

**SCHEME 1:**
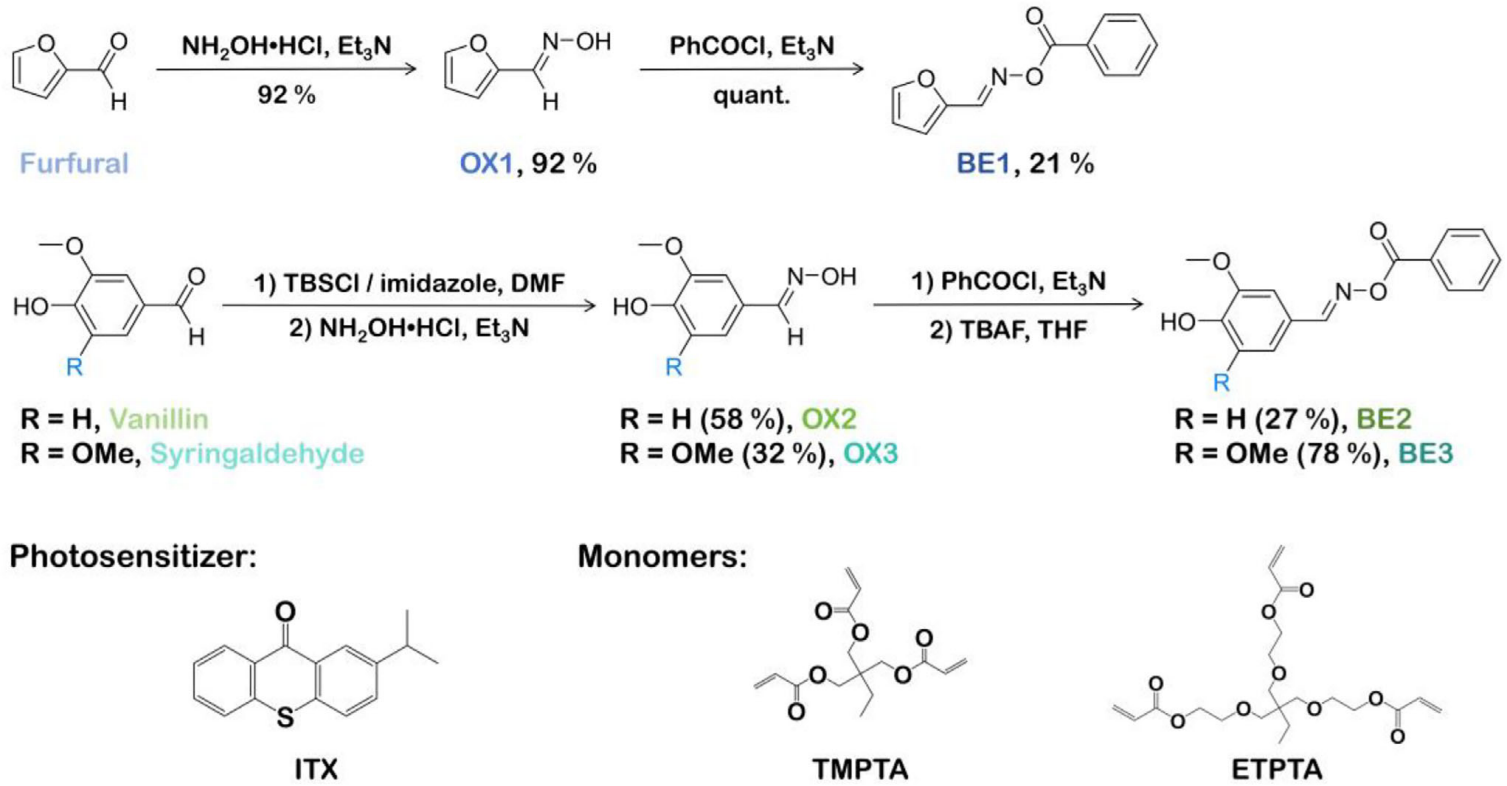
Synthesis of furfural, vanillin, and syringaldehyde‐based benzoyl oxime esters (BEs) and the chemical structures of ITX, TMPTA, and ETPTA.

The synthesis of the furfural‐based benzoyl oxime ester was achieved through a two‐step procedure. In the first step, the oxime (OX1) was prepared by reaction with hydroxylamine hydrochloride in the presence of triethylamine. The second step involved the acylation of the oxime to afford the corresponding benzoyl oxime ester BE1 using benzoyl chloride. For the BE2 and BE3, derived from vanillin and syringaldehyde, respectively, a similar synthetic pathway was employed; however, an additional phenolic protection step was required using TBSCl. The target oxime esters were subsequently obtained after deprotection of the TBS groups with TBAF (Scheme 1).

### 3.2 UV–vis Absorption of BEs

**FIGURE 1:**
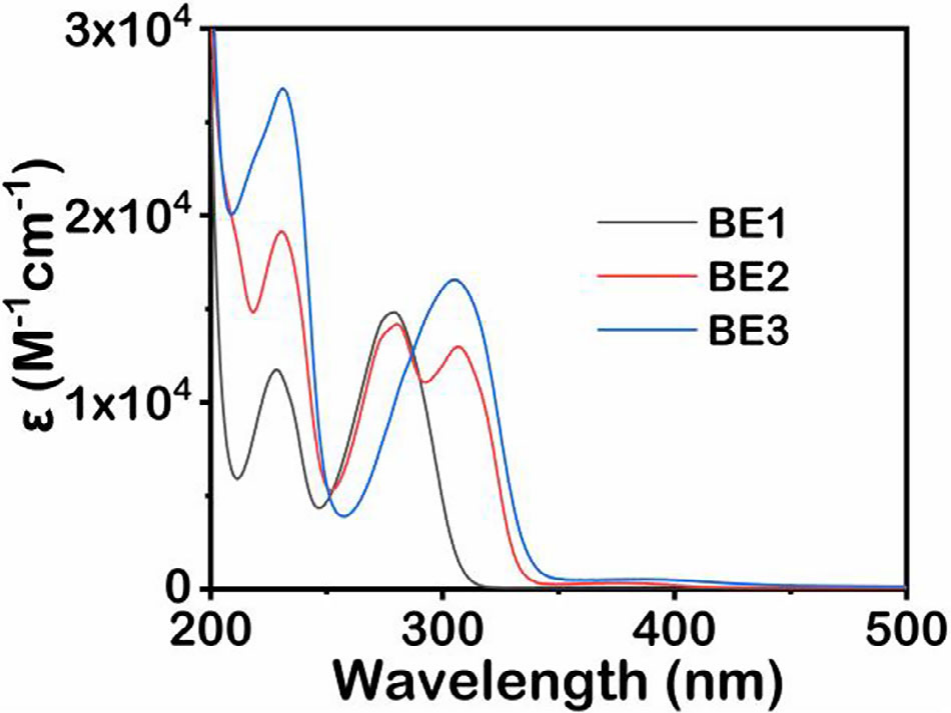
UV–vis absorption spectra of BEs in acetonitrile.

The electronic absorption profiles of BE1‐BE3 were recorded in acetonitrile and are presented in Figure 1. All three oxime esters display intense π→π* bands strictly confined to the near‑UV region [22]; their maximum absorption wavelengths are 279 nm (BE1), 281 nm (BE2) and 305 nm (BE3), with corresponding molar extinction coefficients of 1.48 × 10^4^, 1.41 × 10^4^ and 1.65 × 10^4^ M^−1^cm^−1^, respectively. Despite their respectable extinction coefficients in the UV, BE1‐BE3 are essentially transparent beyond 350 nm (Figure 1). Consequently, when used as stand‑alone photoinitiators, they cannot harvest the Visible light photons (λ > 400 nm) delivered by modern LED or sunlight sources, making visible‑light‑induced polymerization intrinsically inefficient.

Polymerization trials confirm this limitation (Figure S1). At a practical loading of 2 × 10^−5^ mol/g, none of the three BEs induced measurable curing of either TMPTA or ETPTA under 365 or 405 nm LEDs (Figure S1b,c,e,f). Conversions remained below 3 % after 600 s of irradiation, and no gel formation was observed. In addition, BE3 exhibits poor solubility in TMPTA (Figure S1a), further leading to its inefficiency [23]. These observations underscore that visible‑light‑driven radical generation with isolated BEs is practically unattainable. Taken together, these photophysical and performance constraints rationalize why the in situ generation of initiating radicals under visible light is severely limited for isolated BEs molecules. Accordingly, we now employ quantum‑chemical calculations to explore energetically feasible pathways that can liberate initiating radicals from the BEs scaffold under visible‑light irradiation.

### 3.3 Photosensitization Interaction Between BEs and Photosensitizers

#### 3.3.1 Energy Transfer

To evaluate whether visible‑light photosensitization can unlock radical formation from BEs, we computed the key thermodynamic parameters summarized in Table 1. For all three BEs, the singlet‑excited state (S_1_) lies > 40 kcal/mol above the N─O bond dissociation threshold, giving highly exergonic cleavage (ΔH_cleavage S1_ = −43.7 to −52.7 kcal/mol). Although the triplet‑excited state (T_1_) is lower in energy, it is still higher than the bond dissociation energy (BDE) of the N─O bond, rendering N─O homolysis exothermic on T_1_ as well (ΔH_cleavage T1_ = −6.1 to −12.8 kcal/mol). Hence, once a BE molecule is promoted to either S_1_ or T_1_, radical generation via α‑cleavage is thermodynamically favorable [24]. However, as demonstrated in Section 3.2, the individual BEs absorb negligible visible light, making it difficult for them to access these excited states and thus to generate radicals efficiently on their own [25].

**TABLE 1:** Parameters related to BEs and photosensitizers. BDE and E_T_ were obtained from our DFT calculations (see Part 2.11).

Samples	BE1	BE2	BE3	ITX	BP	CQ	DBA
BDE(N─O) (kcal/mol)	43.1	43.1	43.0				
E_S1_ (kcal/mol)	95.8	87.2	86.7	73.8	77.5	58.1	70.2
ΔH_cleavage S1_ (kcal/mol)	−52.7	−44.1	−43.7				
E_T1_ (kcal/mol)	49.2	52.9	55.8	63.8	63.7	43.3	38.2
ΔH_cleavage T1_ (kcal/mol)	−6.1	−9.8	−12.8				

Inspired by the pervasive success of energy‐transfer paradigms, ranging from Förster‐mediated FRET biosensors to Dexter‐controlled triplet harvesting in state‐of‐the‐art OLEDs [26], we envisaged that the dormant reactivity of oxime esters could likewise be unlocked through a photosensitization strategy. Specifically, we introduce a visible‐light‐absorbing photosensitizer (PS) that forms a two‐component photoinitiating system with BE. Upon illumination, the PS is excited and subsequently transfers its triplet energy to the BE via Dexter‐type exchange, thereby populating the BE T_1_ state that is thermodynamically predisposed to N─O homolysis. This design circumvents the intrinsic UV‐limited absorption of the BEs and paves the way for efficient radical generation under visible LEDs or even sunlight.

The energetic landscape summarized in Table 1 further reinforces this assessment. The singlet energies of BE1–BE3 (86.7–95.8 kcal/mol; from Figure S2) exceed those of the visible‐light photosensitizers (58.1–77.5 kcal/mol), giving ΔE_S1_> 0 and making any S_1_(PS) to S_1_(BEs) transfer thermodynamically uphill. Consistent with this, Figure S3 shows negligible overlap between PS emission and BEs absorption. Förster resonance energy transfer (FRET) is a long‐range dipole–dipole process between singlet states whose efficiency depends most critically on the emission–absorption spectral overlap and decays with distance [27]. The lack of overlap, together with ΔE_S1_> 0, thus renders FRET ineffective here, directing attention to Dexter triplet–triplet exchange.

**SCHEME 2:**
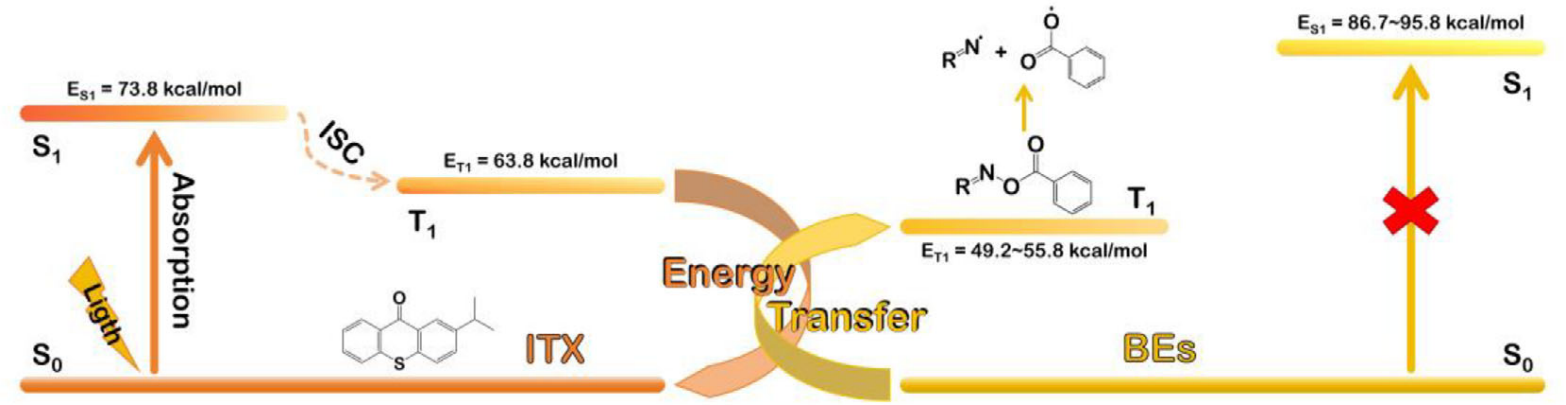
Proposed mechanism of light‐induced energy transfer and oxime ester cleavage to generate free radicals.

Dexter triplet energy transfer is a short‐range, electron‐exchange process that requires transient orbital overlap between an excited donor and a ground‐state acceptor [28]. Its probability decays exponentially with intermolecular distance and therefore follows diffusion‐controlled encounter kinetics [29]. Unlike FRET, which relies on singlet states and spectral overlap, Dexter transfer is largely insensitive to emission–absorption overlap and is most effective for triplet→triplet exchange when the thermodynamic condition E_T1_(donor) > E_T1_(acceptor) is met [30]. Taking ITX as a representative donor‐acceptor pair illustrates the alternative Dexter energy transfer scenario. While its singlet energy lies below that of the BEs, the triplet energy of ITX (63.8 kcal/mol) exceeds the corresponding values of BEs (49.2–55.8 kcal/mol), yielding a negative ΔE_T1_ (−8.0 to −14.6 kcal/mol), which fulfils the requirement for Dexter electron‐exchange‐driven triplet–triplet energy transfer. As depicted in Scheme 2, ITX absorbs light to reach its singlet state and undergoes intersystem crossing to the triplet state [31]. Moreover, the comparatively long phosphorescence lifetime of ^3^ITX affords a sufficient temporal window for diffusive encounters with the oxime esters [32]; a downhill T_1_ (ITX) to T_1_ (BEs) step can populate the reactive triplet state of the BEs, from which exergonic N─O homolysis proceeds. Such an energetic match strongly suggests a robust energy‐transfer interaction between ITX and the oxime ester in the envisaged two‐component photoinitiating systems.

**FIGURE 2:**
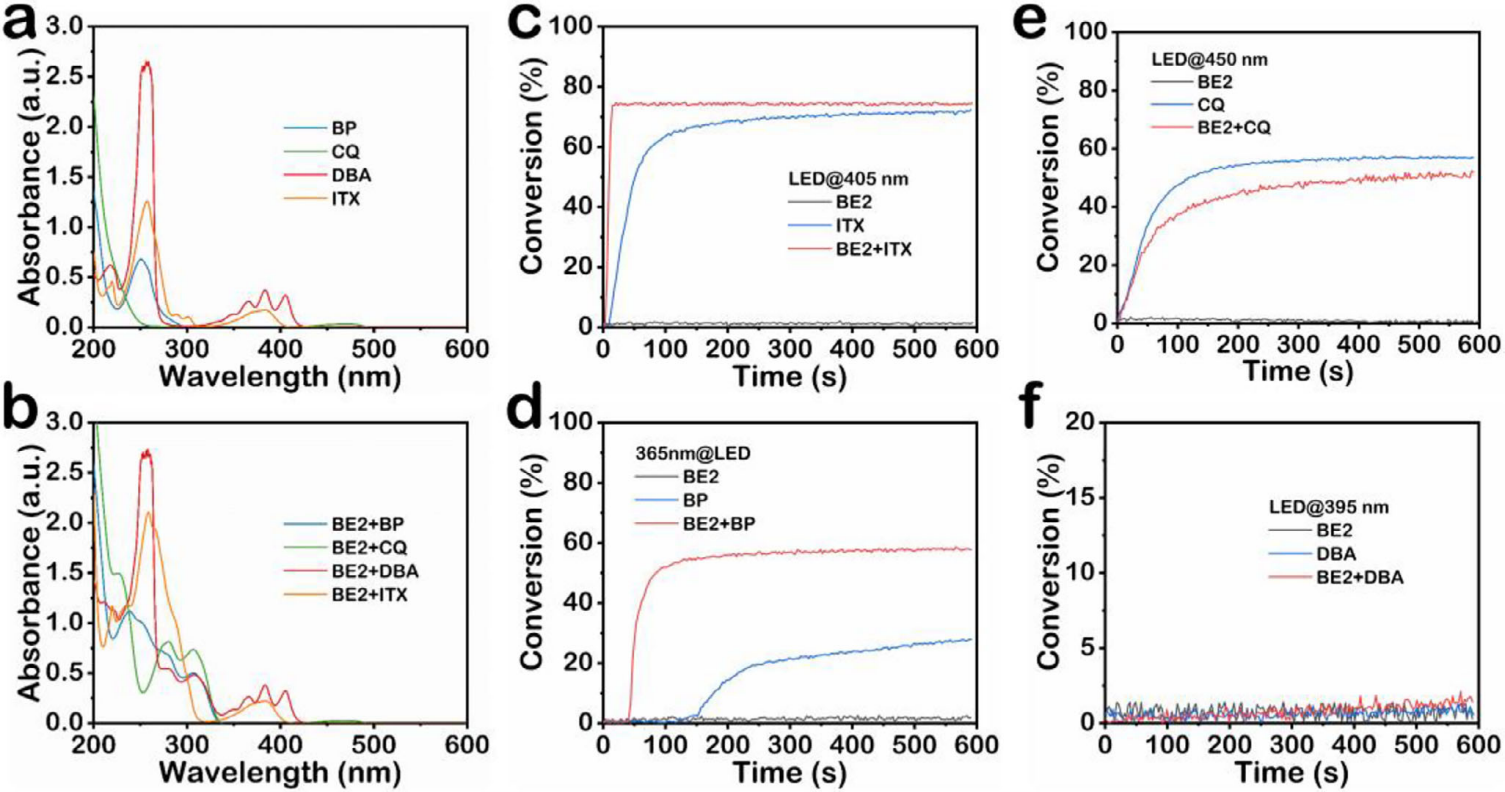
(a) UV–vis absorption spectra of different photosensitizers (BP, CQ, DBA, and ITX; 5 × 10^−5^ M) in acetonitrile, (b) UV–vis absorption spectra of two‐component systems composed of BE2 and each photosensitizer in acetonitrile (5 × 10^−5^ M BE2+ 5 × 10^−5^ M photosensitizer). And photopolymerization of TMPTA thick samples (∼1.5 mm) initiated by two component systems containing BE2 (2 × 10^−5^ mol/g) and photosensitizers (2 × 10^−5^ mol/g) under different LED irradiations: (c) LED@405 nm (BE2+ITX), (d) LED@365 nm (BE2+BP), (e) LED@450 nm (BE2+CQ), and (f) LED@395 nm (BE2+DBA).

To probe whether the energetic criteria outlined above can translate into productive radical generation, we examined BE2 in combination with each photosensitizer under LED irradiation (Figure 2). The UV–vis spectra of the two‐component systems (Figure 2a,b) reveal that adding a PS extends the light absorption to the 360–450 nm window that BE2 alone fails to cover, confirming enhanced photon harvesting in the visible range. The curing kinetics of thick TMPTA samples provide a direct measure of photoinitiation efficiency (Figure 2c–f). Consistent with the Dexter triplet energy‑transfer mechanism predicted above, only those systems where the donor triplet lies above that of BE2, namely BE2+ITX (63.8 kcal/mol) and BE2+BP (63.7 kcal/mol), deliver markedly faster polymerization rates and higher final conversions than either BE2 or the PS alone. By contrast, two‑component photoinitiating systems comprising DBA (38.2 kcal/mol) or CQ (43.3 kcal/mol), whose triplet energies are below that of BE2 (52.9 kcal/mol), display no improvement in curing relative to either component alone under otherwise identical conditions. These outcomes underscore that energy transfer from the PS to the BE2 is the decisive trigger for N─O homolysis and subsequent radical polymerization.

#### 3.3.2 Electron Transfer

To complement the energy transfer analysis, we evaluated whether a photo‑induced electron transfer between BE2 and the visible‑light photosensitizers could account for the photoinitiation behavior. Cyclic voltammetry of BE2 in acetonitrile (Figure S4a) reveals reversible oxidation and reduction waves at E_ox_ = +1.56 eV and E_red_ = −1.54 eV vs. SCE, respectively (Table 2). The Gibbs free‑energy change (ΔG_ET_) for photoinduced electron transfer was estimated with Equation (5). Two electron transfer scenarios were considered (Figure S4b), and the calculated ΔG_ET_ values of BE2 with ITX and BP, the two photosensitizers that display the better photopolymerization performance, are summarized in Table S1. All four ΔG_ET_ values are positive (ΔG_ET_ > 0), meaning that every examined electron transfer pathway is thermodynamically uphill and hard to proceed spontaneously [33]. Accordingly, the improved photopolymerization observed for the BE2‑based two‑component photoinitiating systems is attributed to Dexter‑type triplet energy transfer rather than electron transfer.

**TABLE 2:** Redox potentials and computed excited‐state energy (E^*^) and energy levels of LUMO and HOMO orbitals for different compounds.

	E_ox_ (V)	E_red_ (V)	E* (eV)	HOMO (eV)	LUMO (eV)
BE2	1.56	−1.54		−6.62	−1.13
ITX	1.50 [34]	−1.57 [34]	2.77	−6.10	−1.74
BP	2.95 [35]	−1.78 [36]	2.76	−6.97	−1.62

DFT‑calculated frontier orbitals (Figure S4c) further corroborate this conclusion. The sizeable HOMO‐LUMO gap of BE2 (5.49 eV) renders light absorption energetically costly; the photosensitizers’ relatively lower‑lying LUMO levels prevent formation of a favorable high‑energy electron/low‑energy acceptor pair [37], and the two molecules’ orbitals reside on spatially distant fragments with negligible overlap, perhaps depriving the system of the long‑range electronic coupling required for efficient electron transfer [38]. Overall, the electrochemical data and frontier‑orbital analysis confirm that photo‑induced electron transfer between BE2 and either ITX or BP is both thermodynamically and electronically disfavored. Therefore, the marked enhancement realized in the BE2+PS two‑component photoinitiating systems is attributable to the energy transfer.

#### 3.3.3 Photolysis and Quenching

**FIGURE 3:**
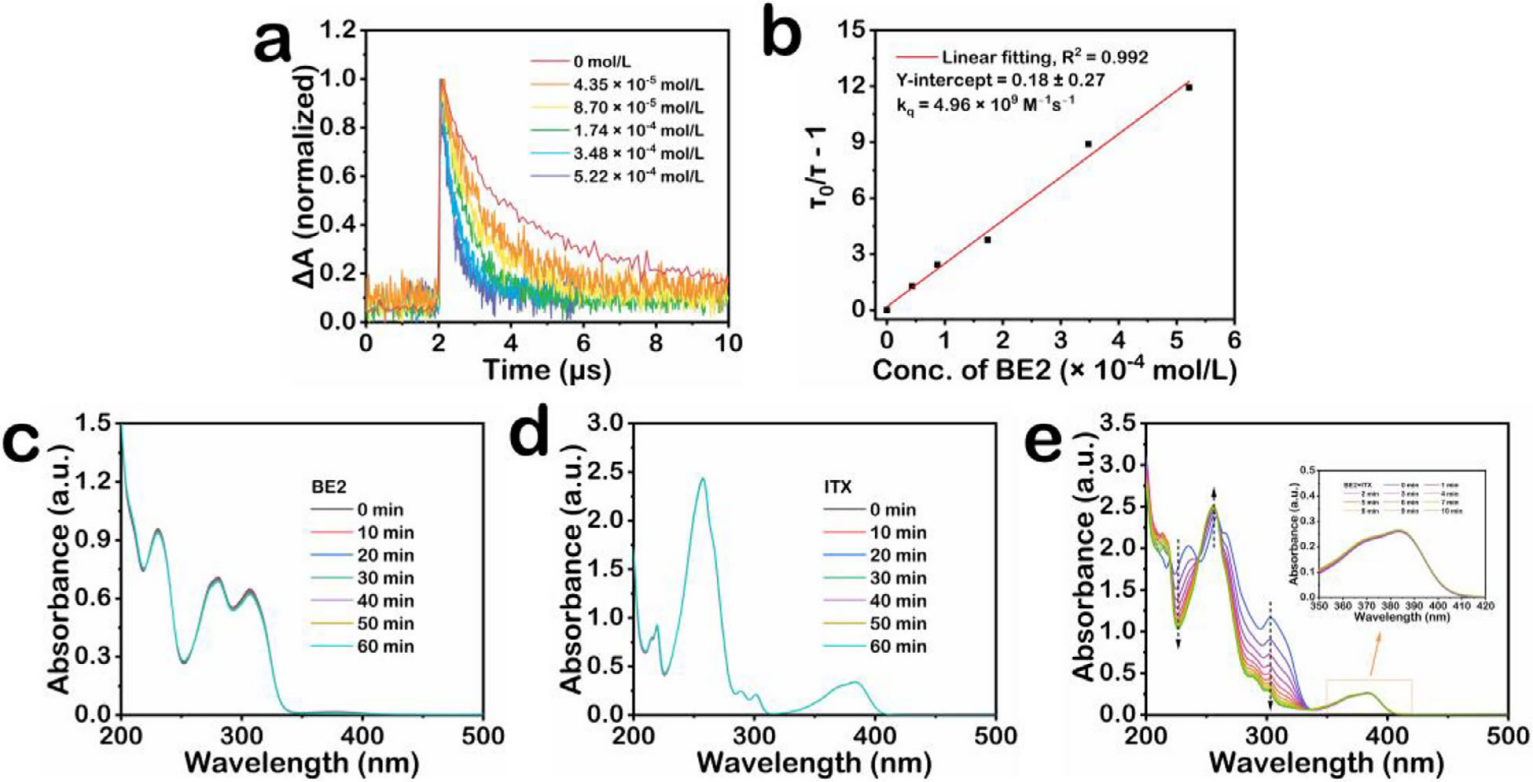
(a) Kinetics corresponding to the transient absorption of ^3^ITX at 600 nm, which is quenched upon subsequent addition of different amounts of BE2, (b) Stern−Volmer treatment for the ^3^ITX quenching, and the steady‐state photolysis of (c) BE2, (d) ITX, and (e) BE2+ITX under LED@405 nm irradiation.

To clarify the interaction between BE2 and ITX and to support the proposed triplet–triplet energy transfer pathway, we monitored the photophysical and photochemical behavior of BEs+PS systems. Incremental addition of BE2 to an ITX solution led to a linear Stern‐Volmer plot with a k_SV_ = 7.7 M^−1^ (Figure S5). This linear relationship indicates dynamic fluorescence quenching and suggests that BE2 interacts with photoexcited ITX through collisional encounters rather than through a dominant pre‐formed ground‐state complex [39]. Because the fluorescence of ITX originates from its S1 state, the steady‐state fluorescence quenching should not be regarded as direct evidence for Dexter‐type triplet–triplet energy transfer. Instead, it provides supporting evidence for excited‐state interactions and frequent collisional encounters between ITX and BE2, which are compatible with the short‐range bimolecular contact required for Dexter‐type energy transfer [40]. The time‑correlated single‑photon counting (TCSPC) shows no measurable change in the native fluorescence lifetime (< 1.4 ns, below the instrumental response) of the BEs themselves (Figure S6), attributed to rapid non‑radiative deactivation pathways of the n→π* oxime chromophore [41], the quenching is attributed to excited‑state interactions initiated on ITX. Upon 355 nm excitation, the transient absorption of the triplet state of ITX monitored at 600 nm [42] shows a mono‐exponential decay with a lifetime τ_0_ = 4653 ns in degassed acetonitrile. Progressive addition of BE2 accelerates the decay without altering the trace shape, consistent with dynamic bimolecular quenching of ^3^ITX (Figure 3a). A lifetime Stern–Volmer analysis of (τ_0_ / τ − 1) vs. [BE2] affords a straight line with an intercept of 0.18 ± 0.27, statistically indistinguishable from zero, indicating dynamic quenching dominates with negligible static contribution (Figure 3b) [43]. The extracted bimolecular quenching constant k_q_ = 4.96 × 10^9^ M^−1^s^−1^ approaches the diffusion limit in acetonitrile, indicating dynamic, encounter‐controlled quenching rather than the formation of a ground‐state complex. Therefore, although fluorescence quenching is treated only as supporting evidence, the LFP triplet‐lifetime quenching provides the principal kinetic evidence for efficient quenching of 3ITX by BE2 and supports Dexter‐type triplet–triplet energy transfer as the main sensitization pathway.

Next, the steady‑state photolysis behavior of different components was examined. Neither BE2 nor ITX alone shows appreciable spectral evolution when exposed to a 405 nm LED for 60 min (Figure 3c,d), and analogous stability is observed for BE1 and BE3 (Figure S7a,b). This rules out significant self‑sensitized decomposition pathways under the experimental conditions. In sharp contrast, BEs+ITX combinations exhibit pronounced steady‑state photolysis. A progressive decrease of the characteristic BEs π→π^*^ absorption is observed (Figure 3e; Figure S7c,d), consistent with the initial N─O bond scission of the oxime ester, leading to an acyloxy radical intermediate [44]. Importantly, the ITX‑derived absorption feature centered at 383 nm, introduced upon photosensitizer addition, remains essentially unchanged throughout irradiation, indicating that the photosensitizer is not consumed but acts catalytically [45]. The linear Stern‐Volmer behavior, the photostability of ITX, and the selective depletion of BEs absorption collectively support a Dexter‑mediated triplet energy transfer from ^3^ITX to BEs, rather than a photo‑induced electron transfer pathway, which would necessarily oxidize or reduce the photosensitizer and alter its chemical structure. These findings close the mechanistic loop: ITX harvests 405 nm photons, transfers its triplet energy to the BEs, and repeatedly participates in the initiation cycle without undergoing irreversible photolysis.

#### 3.3.4 Photosensitization Mechanism

The complete photosensitization cycle operating in the BEs+ITX two‐component photoinitiation system is outlined in Scheme 2. Because BE1–BE3 absorb only in the UV, they cannot be excited directly under visible LEDs. In contrast, ITX exhibits an absorption peak at 383 nm and extends its absorption tail beyond 400 nm, enabling efficient capture of visible light photons. Following photon absorption, ITX undergoes rapid intersystem crossing (ISC) from its first singlet excited state to the lowest triplet state. Energy transfer from ^3^ITX* to the oxime ester proceeds by Dexter exchange because its triplet energy exceeds that of the oxime ester, whereas the opposite ordering is observed for their singlet energies, an energetic relationship that precludes singlet energy transfer. Meanwhile, the calculated positive ΔG_ET_ values indicate that electron transfer between excited ITX and the BEs is thermodynamically unfavorable. Consistently, no clear SET‐related evidence, such as the formation of persistent radical‐ion species or pronounced ITX consumption, was detected during the photochemical studies.

In contrast, laser flash photolysis showed efficient dynamic quenching of the triplet state of ITX by BE2, with a quenching rate constant approaching the diffusion limit. This LFP triplet‐lifetime quenching provides the principal kinetic evidence for the involvement of the triplet state of ITX. When combined with the favorable triplet‐energy alignment, the positive ΔG_ET_ values for electron transfer, and the absence of clear SET‐related signatures, these results support Dexter‐type triplet–triplet energy transfer as the dominant activation pathway in the BEs+ITX system. Once promoted to its T_1_ state, the BEs undergo exergonic N─O bond cleavage (ΔH_cleavage T1_< 0), yielding free radicals. This step is evidenced by the selective depletion of BEs absorption bands during steady‑state photolysis. Because only energy, rather than an electron, is transferred, ITX returns to its ground state unaltered and can re‑enter the cycle, consistent with its unchanged 383 nm signature throughout irradiation.

### 3.4 Photoinitiation Mechanisms

#### 3.4.1 Decarboxylation Reaction

**FIGURE 4:**
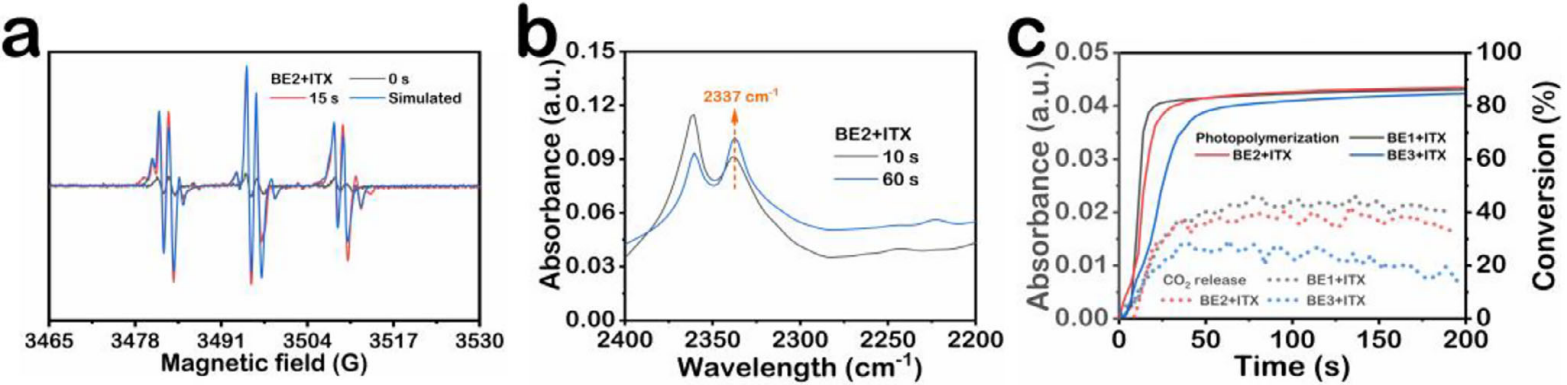
(a) ESR spectra of BE2+ITX photoinitiating systems, (b) FTIR spectra of 2 × 10^−5^ mol/g BE2 + 2 × 10^−5^ mol/g ITX initiation system of thin sample at 10 s and 60 s and (c) absorption intensity of CO_2_ release and conversion curves for different initiation systems (2 × 10^−5^ mol/g BEs + 2 × 10^−5^ mol/g ITX).

Visible‑light activation of the oxime ester ultimately relies on cleavage of the acyloxy radical and its rapid decarboxylation to generate the propagating alkyl radical. To verify this sequence, we combined spin‑trapping ESR and in‑situ FTIR CO_2_ monitoring. Under 405 nm irradiation, BE2 yields the characteristic three‑line spectrum of an acyloxy radical (Figure S11a). However, this signal was very weak when plotted using the same y‐axis range as that used for the BE2+ITX system in Figure 4a. This result indicates that BE2 can undergo limited direct activation under the highly sensitive ESR conditions, but the radical flux generated by BE2 alone is too low to efficiently initiate polymerization in the monomer formulation. This observation is consistent with the negligible photolysis and poor polymerization performance of BE2 alone under 405 nm irradiation. ITX displays a persistent triplet‑derived signal whose intensity remains constant over 60 s (Figure S11b). This behavior indicates a rapid steady state between radical formation and back‑recombination and confirms that ITX cannot sustain a productive radical flux on its own. In the BE2+ITX two‑component photoinitiating system, radical signals emerge immediately after light exposure, and Figure 4a shows the spectrum collected at 15 s as a representative example. Simulation with a_N_ = 13.2 G and a_H_ = 1.5 G identifies the radical as benzoyloxyl radicals (PhCOO·), [46] the acyloxy species expected from N─O homolysis of the oxime ester structure. The continuous growth of this signal indicates a stable and higher radical flux, in line with the rapid polymerization observed for the BE2+ITX system. To compare a successful energy‑transfer system with an unsuccessful one, we next examined CQ as an alternative photosensitizer. Neither CQ nor BE2+CQ gives discernible ESR lines under identical conditions (Figure S11c,d), reaffirming that the absence of a favorable triplet‑energy gap cannot promote radical generation.

Thin films analyzed by in‑situ FTIR display an increasing absorption at 2337 cm^−1^, the asymmetric stretch of CO_2_. This peak grows for BEs+ITX formulations (Figure 4b; Figure S12a,b) but remains unchanged for ITX alone (Figure S12c). Plotting CO_2_ intensity alongside conversion (Figure 4c) shows that the onset of radical polymerization parallels CO_2_ release, confirming that decarboxylation of the benzoyloxyl radical, which liberates CO_2_ and generates the phenyl radical (Ph·), is tightly coupled to the onset of polymerization [47]. Taken together, the ESR and FTIR data outline a coherent mechanism in which Dexter triplet energy transfer from ITX populates the T_1_ state of the oxime ester, N─O bond homolysis yields PhCOO·, and rapid decarboxylation produces the phenyl radical (Ph·) that initiates polymerization. Formulations that lack either a suitable triplet energy donor (BEs alone) or an adequate triplet‑energy gap (CQ) do not generate sufficient radical flux for macroscopic curing.

#### 3.4.2 Photochemical Mechanism of BEs+ITX Two Component Photoinitiating Systems

**SCHEME 3:**
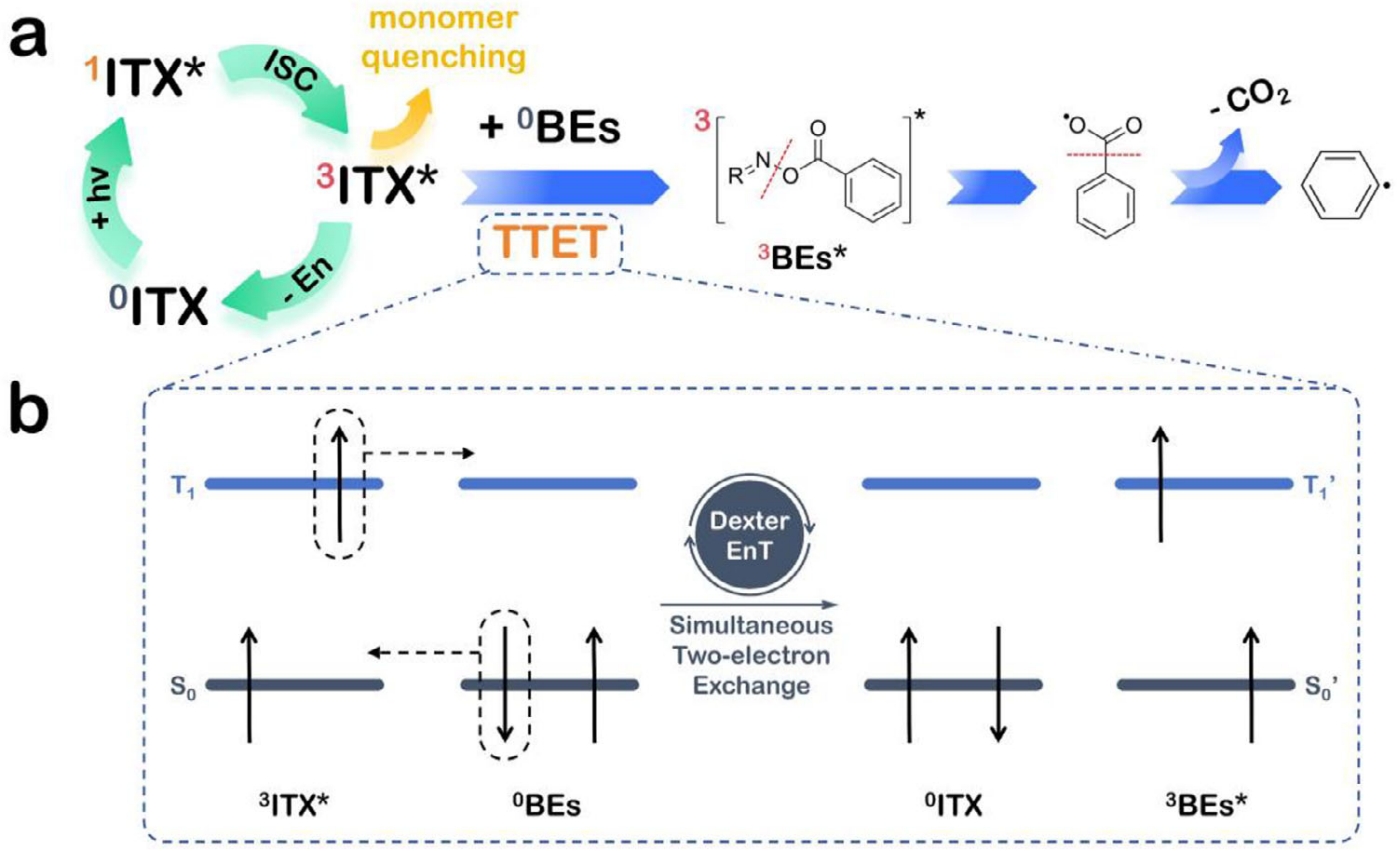
Proposed mechanism of (a) sensitization of ITX to BEs, leading to oxime ester homolysis and radical generation, and (b) triplet‐triplet energy transfer (TTET) process between ITX and BEs.

Scheme 3a integrates the spectroscopic observations with the calculated energetics. Photoexcitation converts ground‑state ITX to its singlet state, followed by efficient intersystem crossing that furnishes the long‑lived triplet ^3^ITX. In the absence of a suitable acceptor, this triplet is predominantly quenched by the acrylate monomer, explaining the slow onset seen for neat ITX. When a BEs molecule is present, however, the favorable ordering E_T1_(ITX) > E_T1_(BEs) enables Dexter‑type triplet–triplet energy transfer that outruns monomer quenching. After the ^3^ITX is generated, short‐range, double‐electron exchange‐mediated transfer delivers its triplet energy to a ground‐state BEs, which are scarcely photoexcitable (Scheme 3b). In Dexter TTET, the electron exchange comprises two simultaneous, symmetry‐equivalent hops. One electron moves from an excited orbital of the donor to the acceptor, and the other moves from an occupied orbital of the acceptor to the donor. This leaves the total charge on both partners unchanged, with only the triplet spin/excitation transferred [48]. The Dexter step requires transient orbital overlap and is only efficient when the triplet energies of the photosensitizer and photoinitiator match. Crucially, the TTET process involves no net electron flow, so the redox states of both partners are preserved. In contrast, SET would move an electron between donor and acceptor, generating radical ions and therefore depends on redox potentials with ΔG_ET_ < 0, typically leaving chemical fingerprints (like new absorption bands). In our system, the uphill singlet energetics, positive ΔG_ET_, and unchanged ITX absorption argue against SET and are consistent with Dexter‐driven TTET as the operative route. The resulting ^3^BEs after TTET undergoes N─O homolysis to give the benzoyloxyl radical identified in the ESR experiment. Rapid decarboxylation of this acyloxy species releases CO_2_, as captured by the growing 2337 cm^−1^ band in the FTIR trace, and simultaneously generates the phenyl radical Ph·. This carbon‑centered radical adds to the acrylate double bond and triggers the chain process that builds the polymer network.

The Dexter‐sensitization strategy uncovered here is not idiosyncratic to BEs+ITX but, rather, offers a transferable design rule for oxime‐ester photoinitiators prepared from the vast and bio‐abundant family of aldehydes. Aldehydes can be converted in one or two steps into oxime esters that are intrinsically photoactive yet typically poor absorbers in the visible, limiting their utility under safer visible light. Our results suggest a straightforward way to overcome this constraint. First, compute or estimate the triplet energy (E_T1_) of the target oxime ester. Then, select a visible‐light photosensitizer with strong absorption in the desired LED/sunlight window and a long‐lived triplet. Critically, ensure that E_T1_(PS) > E_T1_(oxime ester) to guarantee downhill Dexter triplet–triplet energy transfer. Because Dexter exchange is short‐range and diffusion‐controlled, these pairs are readily realized in standard acrylate matrices without covalent linking, enabling modular mixing of bio‐derived oxime esters with off‐the‐shelf sensitizers to extend the usable wavelength range into the visible and deliver fast, high‐conversion curing under low‐irradiance LEDs or sunlight. This general pathway thus opens a practical avenue toward greener, safer, and more sustainable photopolymerization, reducing UV exposure and photoinitiator loadings while leveraging renewable aldehyde feedstocks.

### 3.5 Photopolymerization Performance

**FIGURE 5:**
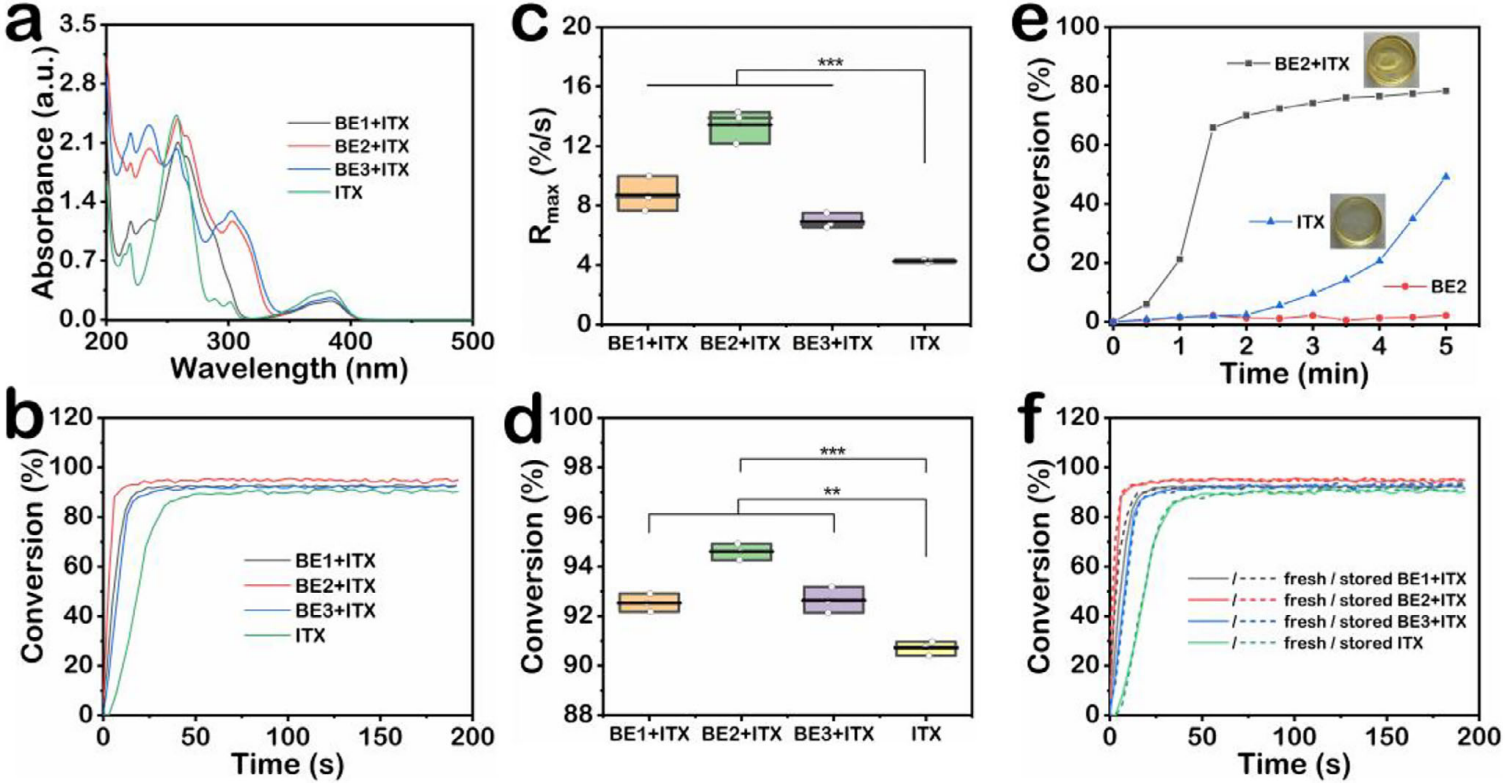
(a) UV–vis absorption spectra of two component photoinitiating systems (5 × 10^−5^ M BEs + 5 × 10^−5^ M ITX) in acetonitrile, (b) photopolymerization kinetics of ETPTA thick samples containing different photoinitiating systems (2 × 10^−5^ mol/g BEs + 2 × 10^−5^ mol/g ITX) under LED@405 nm irradiation, (c, d) maximum polymerization rate (R_max_) and final conversion of ETPTA for different formulations, (e) photopolymerization of ETPTA thick samples of different photoinitiating systems under sunlight irradiation, and (f) polymerization profiles of fresh and stored (in dark room temperature environment for one month) formulations under LED@405 nm. Data in panels (c, d) are presented as box plots from three independent measurements (*n* = 3). Statistical significance was analyzed by one‐way ANOVA. ^**^ *p* < 0.01 and ^***^ *p* < 0.001.

The practical utility of the photosensitized oxime‑ester strategy was assessed in ETPTA, a monomer in which all three BEs dissolve readily (Figure S8a). The BEs+ITX systems exhibit a broadened absorption envelope across the visible region (Figure 5a), confirming that ITX supplementation compensates for the near‑UV limitation of the oxime ester. Upon LED@405 nm irradiation, all BEs+ITX two‐component photoinitiating systems trigger polymerization instantaneously, whereas ITX alone requires approximately a 3 s period prior to starting polymerization (Figure 5b). The comparative kinetics reveal a pronounced rate enhancement (Figure 5c). The BE2+ITX system reaches a maximum polymerization rate (R_max_) of 13.4 ± 1.1 s^−1^, which is more than three times faster than ITX alone (4.3 ± 0.1 s^−1^) (Table S2). After 200 s of irradiation, BEs+ITX systems furnish conversions of 92%–95% (Table S2), with BE2+ITX achieving 94.6 ± 0.3 %, which is statistically higher (*p* < 0.001) than the 90.7 ± 0.3 % obtained with ITX alone (Figure 5d).

Having established superior LED‐driven performance under controlled laboratory conditions, we next evaluated the behavior of the system under natural sunlight. When the best formulation (BE2+ITX) is exposed to midday sunlight, the formulation reaches 78% conversion within 5 min, whereas ITX attains only 49% under identical conditions (Figure 5e). Such rapid progress under an un‑collimated, low‑irradiance solar source (meteorological details in Figure S8b–d) demonstrates the system's outstanding light‑harvesting efficiency and indicates its suitability for cost‑efficient outdoor or large‑area curing applications where conventional high‑power lamps are impractical [49].

Deep through‐cure expands the accessible part thickness and shortens exposure schedules for bulky cross‐sections, thereby improving dimensional fidelity. It also enables curing of weakly transmitting or pigment‐filled resins, where surface‐limited initiator systems typically fail [50]. The BE2+ITX two‐component photoinitiating system exhibits markedly enhanced through‐cure relative to ITX alone. Under 405 nm LED irradiation, the formulation produces a continuously cured column with an observable depth of ∼10 mm, whereas ITX on its own cures only ∼3 mm under identical conditions (Figure S9a, top). Strikingly, the BE2‐sensitised system also remains highly effective under uncollimated sunlight (meteorological details in Figure S9b–d), achieving ∼6 mm cure depth while ITX alone reaches only ∼1 mm (Figure S9a, bottom). These results corroborate that Dexter‐type triplet sensitization of the oxime ester substantially increases the usable penetration window and delivers robust curing even with low‐irradiance, broadband solar light.

Positioning BE2 as a Dexter‐sensitized oxime‐ester initiator against conventional co‐initiators highlights a mechanistic and performance distinction. In thick ETPTA samples under LED@405 nm (Figure S10a), the BE2+ITX system delivers curing kinetics and final conversions that are comparable to those of Iod+ITX, where Iod operates via single‐electron transfer (SET), generating aryl‐radical species, and slightly superior to EDB+ITX, where EDB functions as a hydrogen‐atom‐transfer (HAT) co‐initiator (Figure S10b) [51]. Neither Iod nor EDB alone cures thick samples, underscoring that, in the visible, photosensitizer‐assisted pathways are essential. Overall, BE2, which was sensitized through triplet–triplet Dexter exchange rather than electron or hydrogen transfer, matches the benchmark performance of the SET onium system and marginally outperforms the HAT amine system under identical conditions.

Long‑term stability of photoinitiator formulations is critical for industrial deployment because it guarantees consistent performance during storage and transport [52]. The BEs+ITX photoinitiating systems remain shelf‑stable. After one month of dark storage at room temperature, their polymerization profiles and ultimate conversions are indistinguishable from freshly prepared samples (Figure 5f), underscoring their practical robustness. These observations highlight several practical advantages of the BE2+ITX system. The combination of efficient 405 nm activation, sunlight responsiveness, deep through‐cure and one‐month dark storage stability suggests that this formulation can provide reliable curing under both controlled laboratory irradiation and less standardized ambient‐light conditions. Such properties are particularly attractive for thick coatings, adhesive bonding, large‐area curing, repair coatings, and field‐deployable photopolymerization processes, where high‐power UV lamps are undesirable or impractical. The modular use of a bio‐based oxime ester with a commercial visible‐light sensitizer also provides a convenient formulation strategy for developing more sustainable photoinitiating systems without the need to synthesize strongly visible‐light‐absorbing oxime ester chromophores.

### 3.6 3D Printing Applications

The excellent visible‑light reactivity and shelf stability of the BE2+ITX two‑component photoinitiating system prompted us to explore its suitability for additive manufacturing. A formulation containing 4 × 10^−6^ mol/g BE2 and an equimolar amount of ITX in ETPTA served as the model resin. Real‑time FTIR monitoring of a thick sample (∼1.5 mm) exposed at ∼64 mW/cm^2^ shows > 90% double‑bond conversion within 10 s, confirming that layer doses used in 3D printing are more than sufficient for near‑quantitative cure (Figure S13a). Using a 405 nm direct‑laser‑writing (DLW) setup with ∼1.5 mW/cm^2^ irradiation, we successfully fabricated free‑standing “RUOK” letters within 5 min, with smooth, notch‑free sidewalls and uniform heights (Figure S13b). The letter height is ∼2.7 mm, and the stroke width is ∼1.4 mm. The hollow region at the top of the letter “R” measures ∼0.42 mm in height and shows no unintended bridging (Figure S13c).

**FIGURE 6:**
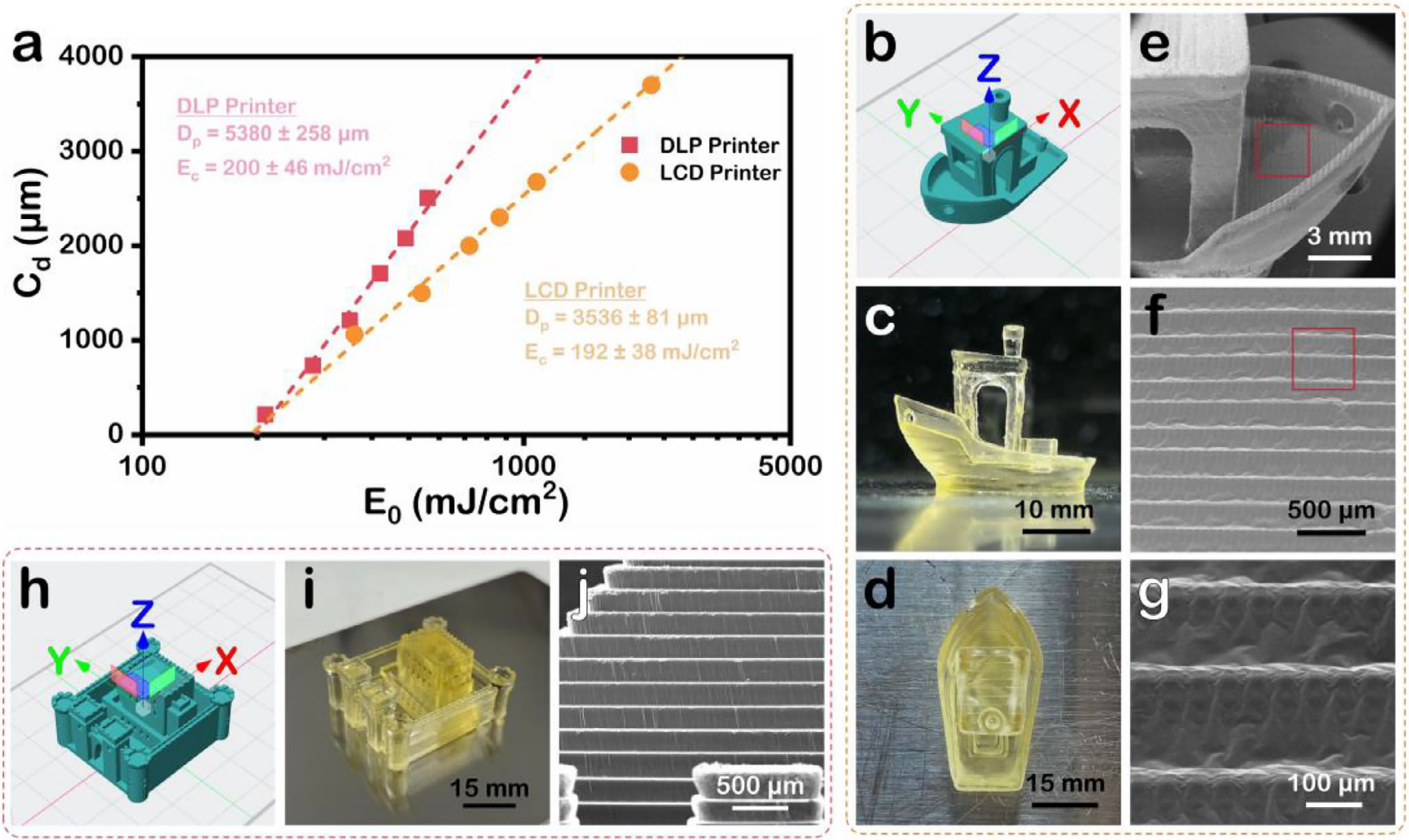
(a) Jacobs working curves with linear fits (for ETPTA containing 4 × 10^−6^ mol/g BE2 + 4 × 10^−6^ mol/g ITX) of DLP printer and LCD printer. The fit parameters and uncertainties are displayed in the plot area. (b) 3D boat model used for LCD printing, (c, d) side and top views of the printed boat, (e–g) SEM images showing structural details at different magnifications, (h) 3D castle model used for DLP printing, (i) castle structures obtained, and (j) SEM image of the printed castle roof.

The Jacobs working curve was tested across different printers to better guide the 3D printing process. For the same BE2+ITX formulation (Figure S13a), Jacobs plots acquired on two 405 nm printers, DLP (∼1.4 mW/cm^2^) and LCD (∼7.2 mW/cm^2^), give similar critical doses E_c_ ≈ 200 mJ/cm^2^ from the linear fits (Figure 6a) [53]. This convergence is expected because E_c_ reflects a chemical threshold, dominated by the initiator/co‐initiator's effective absorption and quantum yield together with early radical losses (e.g., oxygen inhibition). When the excitation wavelength is identical, dose calibration is consistent, and oxygen and post‐processing are well controlled, different light engines tend to yield comparable E_c_. For comparison, the benchmark EDB+ITX formulation was also evaluated on the 405 nm LCD printer under the same resin conditions. The corresponding Jacobs plot remains linear and affords E_c_ = 177 ± 55 mJ/cm^2^ (Figure S13d). The E_c_ value is in the same range as that obtained for BE2+ITX, indicating a comparable chemical threshold for gelation under these conditions. By contrast, the penetration depth differs. D_p_ from the DLP fit is larger than that from the LCD fit, indicating deeper effective light transport with the DLP optics. This is consistent with the more collimated, narrower‐band projection of DLP (reduced angular spread and parasitic filtering), whereas the LCD stack (polarizers, liquid‐crystal panel) introduces additional attenuation and angular divergence, shortening the effective D_p_ [54]. Notably, D_p_ is extracted from the slope of the Jacobs plot and is therefore essentially independent of irradiance, so the higher LCD intensity does not increase D_p_.

The Jacobs relation was further used to set build parameters. For an LCD printer with 7.2 mW/cm^2^, targeting a 0.1 mm layer (C_d_ = 100 µm), giving E_0_ ≈ 204 mJ/cm^2^ corresponds to ∼28.3 s exposure per layer. For a DLP printer with 1.4 mW/cm^2^, targeting a 0.2 mm layer (C_d_ = 200 µm), giving E_0_ ≈ 208 mJ/cm^2^ corresponds to ∼156.2 s exposure per layer. Therefore, for the actual printing process, we selected layer thicknesses of 0.1 mm with an exposure time of 30 s for the LCD printer and layer thicknesses of 0.2 mm with an exposure time of 160 s for the DLP printer, respectively. These settings place C_d_ slightly above the programmed layer height, providing reliable interlayer adhesion while avoiding over‐cure. Adjustments (e.g., longer base‐layer exposure or minor safety factors for pigmented builds) can then be made empirically around this Jacobian baseline.

The formulation was transferred to a consumer‑grade 405 nm LCD printer to fabricate a boat model with internal cavities and portholes. Dimensional fidelity relative to the model design is 102.4% in X, 101.8% in Y, and 100.3% in Z (Figure 6b–d). SEM images reveal printed structure is regular and well‑defined (Figure 6e–g). In‑plane pixel resolution is ∼50 µm (Figure S14a), and the printed layer height is 0.1 mm (Figure S14b), matching the printer settings. To demonstrate platform versatility, the resin was processed on a 405 nm DLP projector. The DLP‐printed castle reproduces the geometry with excellent dimensional fidelity. The measured X, Y, and Z sizes are 99.7%, 101.2%, and 98.5% of the design, respectively (Figure 6h). Macroscopically, key architectural details including gateways, towers, windows, and crenellations are crisply resolved (Figure 6i). SEM further reveals a staircase roof formed by regular layer‐by‐layer stacking with a layer thickness of 200 µm (Figure 6j), identical to the set layer height. This agreement confirms that print parameters derived from the Jacobs working curve provide reliable guidance for matching exposure dose to layer height, ensuring sufficient interlayer bonding while suppressing over‐cure. Notably, edges are sharp, and corners are well defined (Figure S14c,d), with no notches, bulging, or unintended bridging, evidencing effective control of lateral light bleed and radial diffusion. Another pixel‑art cat figurine was fabricated with high fidelity to the jagged‑edge silhouette (Figure S14e,f). The realized dimensions correspond to 98.6% (X), 99.2% (Y), and 98.2% (Z) of the design values, demonstrating remarkably high printing accuracy.

To directly compare printing modalities, we reprinted the same castle model by LCD using the same layer thickness (0.2 mm) but a 35 s exposure per layer. The LCD‐printed part exhibits similarly high fidelity (Figure S14g). The dimensional retention in X, Y, and Z reaches 98.0%, 98.0%, and 97.0%, respectively, and the measured layer thickness matches the 0.2 mm setting (Figure S14h). The most salient difference, seen by SEM, is that the LCD part reveals pixel grids on the X–Y plane (Figure S14i), whereas the DLP part does not (Figure S14d). This discrepancy can be attributed to the fundamental principles of imaging physics. LCD printing operates as a mask near‐contact exposure with a black‐matrix aperture, so pixel edges are directly recorded on the resin [55]. In contrast, DLP relies on projection imaging from a digital micromirror device (DMD), where the optical point‐spread function and built‐in anti‐aliasing effectively low‐pass smooth the pixel lattice [56]. Consequently, LCD preserves pixel boundaries as fine surface texturing, while DLP yields optically blended, visually continuous features despite comparable dimensional accuracy.

This single formulation performs reliably on DLW, LCD and DLP printers, underscoring the breadth of the BE‑sensitized photo‑energy‑transfer strategy. It combines broad compatibility with common 3D printing platforms, cures rapidly, and maintains the high resolution dictated by digital optics. Collectively, these results validate the predictive value of the Jacobs curve and position BE2+ITX as a practical alternative to conventional photoinitiators for 3D printing. Beyond demonstrating printability, the compatibility of one BE2+ITX formulation with DLW, LCD, and DLP platforms suggests broad applicability across both high‐resolution microfabrication and larger‐scale vat photopolymerization. In future applications, this strategy could be extended to customized biomedical devices, cell‐contacting materials, hydrogel printing, microstructured coatings, and sustainable resin systems in which mild visible‐light exposure, reduced UV damage, and good formulation stability are required. The triplet‐energy‐matching principle also offers a predictive framework for adapting other bio‐derived oxime esters to different light engines by selecting sensitizers with suitable absorption and triplet energies.

### 3.7 Biological Safety of Photoinitiating Systems

The biological safety of the photoinitiating systems was first evaluated under dark conditions, since any intrinsic cytotoxicity of the initiators would directly limit their use in cell‐contacting photopolymerization. C_2_C_12_ myoblasts were chosen as a model because they are sensitive to changes in proliferation and morphology, and because they are representative of muscle‐related tissue engineering targets [57]. Cell confluency was monitored in real time for 40 h by phase contrast live cell imaging with automated image analysis, which provides an integrated readout of cell attachment, spreading and growth. The cytocompatibility of the individual photoinitiators ITX, BE2 and EDB at 25 and 50 mM was first examined. In the presence of ITX, C_2_C_12_ cells remained attached and preserved a fibroblast‐like morphology, yet their confluency increased more slowly than in the untreated control. At both 25 and 50 mM, the ITX curves lay below the control curve throughout most of the experiment, and after 40 h, ITX‐treated cultures reached lower confluency values (Figure S15a). These data indicate that ITX exerts a mild, concentration‐dependent inhibitory effect on myoblast proliferation under the chosen conditions. This trend is in line with prior in vitro screening studies reporting that ITX can induce dose‐dependent cytotoxicity across multiple tissue‐derived cell models at micromolar concentrations, and that irradiation can further enhance cytotoxicity in such systems [58]. In contrast, BE2 and EDB did not measurably interfere with C_2_C_12_ expansion. The confluency curves of BE2 and EDB at 25 and 50 mM closely followed that of the control throughout the 40 h recording period, and cultures approached almost complete monolayer coverage (Figure S15b). In the previous literature, EDB shows a dose‐dependent reduction in viability in human pulp‐derived cells with an EC50 of 0.68 mM after 24 h exposure, indicating a moderate intrinsic cytotoxicity that depends on the biological model and test format [59]. These data show that the bio‐based BE2 exhibited good short‐term cytocompatibility in the concentration range relevant for photopolymerization.

**FIGURE 7:**
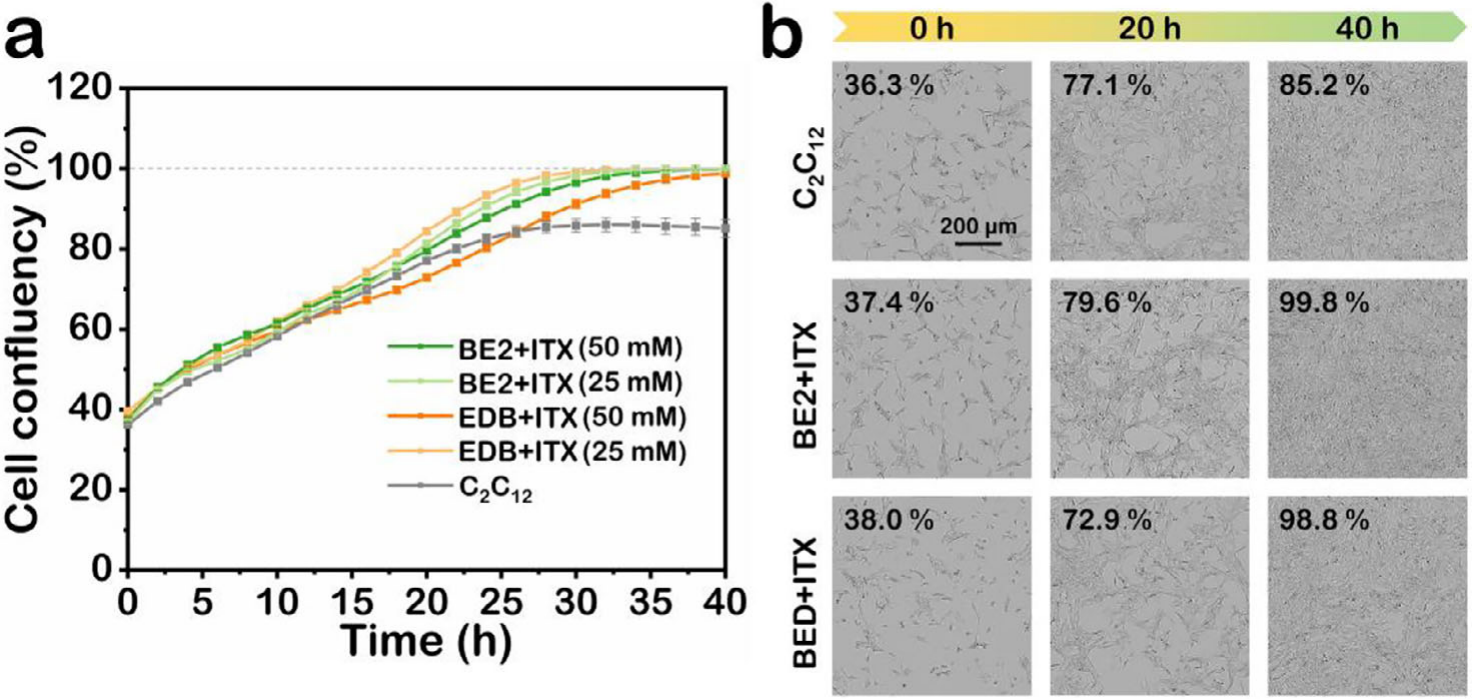
(a) Real‐time cell confluency of C_2_C_12_ myoblasts cultured for 40 h in growth medium containing ITX+BE2 or ITX+EDB at 25 or 50 mM, compared with untreated control cells. (b) Representative phase contrast micrographs of C_2_C_12_ cells exposed to BE2+ITX or EDB+ITX, with BE2 or EDB and ITX each at 50 mM, recorded at 0, 20 and 40 h. The corresponding confluency values are indicated.

The cytocompatibility of the two‑component photoinitiating systems was then examined for combinations of ITX with BE2 or EDB (Figure 7a). For all two‑component systems, C_2_C_12_ cells attached efficiently after seeding and their confluency increased from below 40% at 0 h to more than 80% at 24 h. When BE2+ITX or EDB+ITX were both present at 25 mM, the confluency curves closely followed that of the untreated control throughout the recording period. This result indicates that the incorporation of BE2 or EDB into ITX‐based systems does not compromise cell proliferation at this concentration. At 50 mM for each component, a difference between the two‑component systems became apparent. The confluency curves for ITX+BE2 remained very close to the control curve at all time points and reached values around 100% at 40 h. The curves for ITX+EDB also increased steadily, yet in the mid‐growth phase, they were clearly below the control and below the BE2+ITX curves. Although C_2_C_12_ cells in the EDB+ITX group continued to proliferate and reached high final confluency, this transient depression indicates a somewhat lower cytocompatibility for the EDB‐based two‑component system at the highest concentration when compared with the BE2‐based system, in which cell growth was essentially indistinguishable from the control.

Phase contrast microscopy provided qualitative confirmation of these findings (Figure 7b). At 0 h, C_2_C_12_ cells in control, BE2+ITX, and EDB+ITX conditions exhibited the expected spindle‐shaped or polygonal morphology and were well spread on the substrate. After 20 h, cultures exposed to BE2+ITX contained dense networks of elongated myoblasts that strongly resembled the control, while EDB+ITX cultures also showed extensive spreading and cell‐to‐cell contacts. At 40 h, both BE2+ITX and EDB+ITX groups formed nearly continuous monolayers with confluency values close to 100%, and no widespread rounding, detachment or accumulation of cell debris was observed [60]. The preservation of normal myoblast morphology together with the high endpoint confluency confirms that both two‑component systems are compatible with C_2_C_12_ cells over 40 h. Within this context, the BE2‐based system appears slightly more favorable than the EDB‐based system at 50 mM for each component, consistent with the confluency profiles. These results highlight BE2 as a biologically safe and bio‐based photoinitiator that is suitable for further application in light‐mediated material formation and bio‐fabrication.

## 4 Conclusion

By integrating bio‐based benzoyl oxime esters with a high triplet energy visible light sensitizer, this work establishes a general triplet energy matching Dexter‑type triplet–triplet energy transfer strategy in which 405 nm excited ITX first forms a long‐lived triplet state and then transfers its triplet energy to intrinsically UV absorbing oxime esters so that their reactive triplet states can be accessed under visible light and replace type II activation. Quantum chemical calculations, electrochemistry, LFP, ESR spin trapping, and in situ FTIR consistently show that TTET from ITX to the oxime esters selectively populates their reactive triplet states, while photoinduced electron transfer and hydrogen atom transfer channels remain negligible, so benzoyloxyl cleavage followed by rapid decarboxylation generates phenyl radicals for initiation. This triplet‐sensitized activation enables fast curing, deep through‐cure, and robust performance under low‐energy visible light and natural sunlight, and it delivers high‐fidelity microfabrication on DLW, LCD, and DLP platforms. The BE2‐based two‐component system, built from a bio‐derived oxime ester accessible in only a few steps from simple aldehydes, achieves curing rates and printing accuracy that rival or exceed benchmark onium and amine photoinitiators while maintaining favorable cytocompatibility toward C_2_C_12_ myoblasts. By using triplet energy transfer to activate a bio‐derived oxime ester, it offers a greener and more sustainable alternative to conventional photo‐oxidation–reduction systems that rely on iodonium salts with ITX or hydrogen‐donating amines such as EDB as co‐initiators. Looking ahead, this triplet energy transfer design concept can guide the development of new aldehyde‐derived oxime esters and high‐triplet‐energy sensitizers for low‐energy visible‐light and sunlight‐assisted curing, and may be further translated to stable hydrogel and bio‐based resin platforms for oxygen‐tolerant, large‐area, high‐resolution additive manufacturing and future biofabrication.

## Author Contributions

**Kaidan Yang**: data curation, Writing – original draft. **Gérard Audran**: writing – review and editing, data curation. **Jean‐Patrick Joly**: data curation, writing – review and editing. **Michael Schmitt**: data curation, writing – review and editing. **Jacques Lalevée**: conceptualization, investigation, funding acquisition, methodology, validation, writing – review and editing. **Fabrice Morlet‐savary**: data curation. **Coralie Ohl**: data curation, writing – review and editing. **Jing Zhang**: conceptualization, methodology, validation, investigation, writing – review and editing. **Céline Dietlin**: data curation, writing – review and editing. **Margot Manivel**: data curation. **Pu Xiao**: conceptualization, methodology, validation, investigation, writing – review and editing, funding acquisition.

## Conflicts of Interest

The authors declare no conflicts of interest.

## Supporting information




**Supporting File**: smll74715‐sup‐0001‐SuppMat.docx.

## Data Availability

The data that support the findings of this study are available from the corresponding author upon reasonable request.
