# Supplementary material for: Triplet Energy Transfer Strategy for Visible Light Activation of Bio‐Based Oxime Esters in Photopolymerization and 3D Printing
Source: Small. 2026 Jul 23;22(52):e74715. doi: 10.1002/smll.74715 (PMC13580275; doi:10.1002/smll.74715)
Supplement: Supplementary file 1 [file SMLL-22-e74715-s001.docx]

# Supporting Information

**Triplet Energy Transfer Strategy for Visible Light Activation of Bio-Based Oxime Esters in Photopolymerization and 3D Printing**

Kaidan Yang ^a, b^, Céline Dietlin ^a, b^, Coralie Ohl ^a, b^, Margot Manivel ^c^, Fabrice Morlet-Savary ^a, b^, Michael Schmitt ^a, b^, Gérard Audran ^c^, Jean-Patrick Joly ^c^, Jing Zhang ^d^, Pu Xiao ^e^*, Jacques Lalevée ^a, b, f^*

^a^ Université de Haute-Alsace, CNRS, IS2M UMR7361, F-68100 Mulhouse, France.

^b^ Université de Strasbourg, 4 Rue Blaise Pascal, F-67081 Strasbourg, France.

^c^ Aix Marseille Université, CNRS, ICR UMR7273, 13397 Marseille Cedex 20, France.

^d^ Future Industries Institute, Adelaide University, Mawson Lakes, SA 5095, Australia.

^e^ State Key Laboratory of High Performance Ceramics, Shanghai Institute of Ceramics, Chinese Academy of Sciences, Shanghai 200050, P. R. China.

^f^ Institut Universitaire de France.

## 1. Experimental section

### 1.1. Materials

Trimethylolpropane triacrylate (TMPTA; LAROMER^®^ TMPTA) was obtained from BASF. Ethoxylated trimethylolpropane triacrylate (ETPTA; SR454), 2-isopropylthioxanthone (ITX; SpeedCure 2-ITX), bis(4-tert-butylphenyl) iodonium hexafluorophosphate (Iod; SpeedCure 938), and ethyl 4-(dimethylamino)benzoate (EDB; SpeedCure EDB) were purchased from Arkema Sartomer EMEA. (±)-Camphorquinone (CQ), N-tert-butyl-α-phenylnitrone (PBN), and tetrabutylammonium hexafluorophosphate were obtained from TCI EUROPE N.V. Acetonitrile, benzophenone (BP), and tert-butylbenzene (TBB) were purchased from Sigma-Aldrich. 9,10-Dibutoxyanthracene (DBA) was obtained from INDAGO GmbH. Unless otherwise stated, all materials were used as received without further purification.

### 1.2. Irradiance measurement

The light source was positioned 2 cm above a Thorlabs compact USB power meter (PM16-425) configured for direct irradiance readout. After setting the calibration wavelength and performing a dark/zero correction, the sensor was centered and held normal to the beam. After the system stabilized, the irradiance (mW/cm^2^) was recorded.

### 1.3. UV–Vis absorption and steady-state photolysis

UV–Vis spectra of the photoinitiating systems in acetonitrile were recorded on a JASCO V750. Samples (BEs and ITX, each 5 × 10^−5^ M) were then irradiated with a 405 nm LED (~29 mW/cm^2^), and spectra were collected at defined irradiation time to monitor photolysis.

### 1.4. Photopolymerization

BEs (one-component) or BE+photoinitiators (two-component) were dissolved in TMPTA (or ETPTA) under dark conditions. Thick samples (~1.5 mm) were prepared by dispensing six drops into 13 mm plastic molds; thin samples were sandwiched between two ~25 µm polypropylene films to limit oxygen inhibition. Real-time FTIR (JASCO FT/IR-6600) monitored the acrylate bands at ~6150 cm^−1^ (thick) and ~1650 cm^−1^ (thin). Illumination began 10 s after acquisition using LEDs at 405 nm (64 mW/cm^2^), 450 nm (25 mW/cm^2^), 365 nm (13 mW/cm^2^) or 395 nm (17 mW/cm^2^) LED. Acrylate conversion was calculated as:

$$\begin{aligned} Conversion \left( \% \right)=\left( \frac{A_{0}-A_{T}}{A_{0}} \right)\times100\#\left( 1 \right) \end{aligned}$$

where A_0_ is the initial peak area before light irradiation and A_t_ is the peak area after being irradiated with light for t seconds.

The polymerization rate R_p_ is defined as the rate of disappearance of polymerizable double bonds:

$$\begin{aligned} R_{p}=-\frac{d\left[ M \right]}{dt}\#\left( 2 \right) \end{aligned}$$

where [M] is the concentration of double bonds at time t. Assuming Beer–Lambert behavior, the absorbance is proportional to the double-bond concentration,[1] so

$$\begin{aligned} Conversion \left( \% \right)=(1-\frac{\left[ M \right]}{[M_{0}]})\times100\#\left( 3 \right) \end{aligned}$$

where [M_0_] is the initial concentration of polymerizable double bonds. Differentiating with respect to time gives:

$$\begin{aligned} \frac{dConversion\left( \% \right)}{dt}=-\frac{100}{\left[ M_{0} \right]}\frac{d\left[ M \right]}{dt}=\frac{R_{p}}{\left[ M_{0} \right]}\times100 (s^{-1})\#\left( 4 \right) \end{aligned}$$

showing that $\frac{R_{P}}{[M_{0}]}\times100 (s^{-1})$ corresponds to the instantaneous slope, that is, the first derivative of the double-bond conversion versus time curve. In practice, this quantity is obtained by differentiating the experimental conversion–time data, and the highest value of the slope over the curing period is reported as the maximum polymerization rate (R_max_).

For thin films, CO_2_ release was tracked by integrating the 2337 cm^−1^ band (asymmetric stretch of CO_2_) over time.[2] The areas were baseline-corrected and normalized to 0 at 0 s to compare different photoinitiating systems semi-quantitatively.

Formulation stability was assessed by comparing RT-FTIR polymerization curves of fresh samples with those stored in the dark for 1 month. For sunlight tests, two component systems (BEs+ITX) were applied as thick samples and irradiated outdoors: 30 s sunlight exposure followed by FTIR acquisition, repeated 10 times (total 5 min) to track acrylate conversion.

Silicone tubing (inner diameter ~10 mm) was sealed bottom with Parafilm, filled with the formulations. Samples were irradiated with a 405 nm LED (~64 mW/cm^2^) or direct sunlight for 2 or 5 min. The cured cylinder was demolded, wiped to remove uncured resin, and its height measured with a digital caliper (depth of cure = column height).

### 1.5. Fluorescence measurements

Steady-state emission spectra of molecules in acetonitrile (5 × 10^−5^ M) were recorded on a JASCO FP-750. Fluorescence quenching was evaluated by titrating BE2 into ITX (1 × 10^−4^ M) and monitoring emission (Stern–Volmer analysis). Fluorescence lifetimes were measured on a HORIBA PPD-850 fluorimeter.

### 1.6. Cyclic Voltammetry

Cyclic voltammetry of BE2 was performed in acetonitrile containing 0.1 M TBAPF using an electrochemical workstation (OGF01A, OrigaFlex) under N_2_. The photoinduced electron-transfer free energy was calculated by Rehm-Weller equation as:

$$\begin{aligned} \Delta G_{ET}=E_{ox}-E_{red}-E^{*}+C\#\left( 5 \right) \end{aligned}$$

where E_ox_ and E_red_ are the donor/acceptor redox potentials and E* is the excited-state energy,[3] and C is the coulombic term (C ∼ 0.06 eV in acetonitrile).[4]

### 1.7. Electron Spin Resonance Spin Trapping (ESR-ST)

Radicals under 405 nm LED irradiation were probed on a Bruker EMX-plus (X-band) using TBB as solvent. Analytes were 1 × 10^−3^ M with PBN (5 × 10^−3^ M) as the spin trap, under N_2_ at room temperature. Spectra were simulated with PEST WINSIM program.

### 1.8. Laser Flash Photolysis (LFP)

Transient absorption measurements were carried out on a Q-switched Nd:YAG laser system (355 nm, 3–5 ns pulse width; Continuum Minilite ML II) coupled to a Luzchem LFP-111 detection setup (pulsed Xe probe lamp, monochromator, fast PMT, and digitizer).[5] Experiments were performed at ambient temperature under N_2_. An ITX solution (2 × 10^−4^ M in acetonitrile) was placed in a quartz cuvette and monitored at 600 nm to follow the decay of the ITX triplet state (^3^ITX), and the lifetime in the absence of quencher was recorded as τ_0_. Triplet quenching was evaluated by titrating a BE2 stock solution (1 × 10^−2^ M in acetonitrile) into the ITX solution. After each addition, the mixture was gently mixed and the decay at 600 nm was re-measured to obtain the lifetime τ at the corresponding BE2 concentration [BE2].[6] The lifetime Stern–Volmer relationship:

$$\begin{aligned} \frac{\tau_{0}}{\tau}=1+k_{q}\tau_{0}\left[ BE2 \right]\#\left( 6 \right) \end{aligned}$$

was used to analyze the quenching kinetics. Plots of (τ_0_ / τ − 1) versus [BE2] were fitted by linear regression to obtain the slope k_q_·τ_0_. The bimolecular quenching constant was then calculated as:

$$\begin{aligned} k_{q}=\frac{slope}{\tau_{0}}\#\left( 7 \right) \end{aligned}$$

The intercept was inspected to verify the absence of a static component. All fits report means values with their standard errors (or 95 % confidence intervals, as indicated).

### 1.9. Jacobs working curve

At room temperature, identical formulation (BE2 and ITX, each 4 × 10^−6^ mol/g in ETPTA) were photocured on an Anycubic Photon D2 (DLP, 405 nm, ~1.4 mW/cm^2^) and an Anycubic Photon Mono (LCD, 405 nm, ~7.2 mW/cm^2^). The liquid formulation was dispensed into a glass well (base 2 × 2 cm, height 0.5 cm) and exposed to the printer irradiation for 50–400 s. After curing, excess resin was removed with ethanol, samples were dried, and the cured thickness was measured three times per exposure with a digital indicator (ID-C112XB Mitutoyo), the mean value was taken as the cure depth C_d_. Radiant exposure (E_0_) was calculated as:

$$\begin{aligned} E_{0}=I\times t\#\left( 8 \right) \end{aligned}$$

where I is the irradiance and t is the exposure time. Data were fitted to the Jacobs working curve:

$$\begin{aligned} C_{d}=D_{p}\times\ln\left( \frac{E_{0}}{E_{c}} \right)=D_{p}\ln\left( E_{0} \right)+b\#\left( 9 \right) \end{aligned}$$

where the slope gives the penetration depth D_p_ of the formulation and the x-intercept yields the critical exposure E_c_.[7]

### 1.10. 3D Printing

Two component ETPTA formulation (BE2 and ITX, each 4.0 × 10^−6^ mol/g) were used for direct laser writing with a 405 nm diode (~1.5 mW/cm^2^, Thorlabs). The same formulations were 3D-printed at 405 nm on a DLP 3D printer (~1.4 mW/cm^2^, Anycubic Photon D2; 0.20 mm layers, 160 s per layer) and an LCD 3D printer (~7.2 mW/cm^2^, Anycubic Photon Mono; 0.10 mm layers, 30 s per layer). Printed parts were imaged optically, and their dimensions were measured by numerical optical microscopy (Olympus DSX-HRSU), followed by SEM examination.

### 1.11. Computational procedure

Density functional theory (DFT) calculations were performed in Gaussian 09. Ground-state geometries were optimized at the UB3LYP/6-31g(d) level, and single-point energies for frontier orbitals were computed at MPW1PW91/6-31g(d). The following quantities were obtained: bond dissociation energy (BDE), triplet state energy (E_T1_), highest occupied molecular orbital (HOMO), and lowest unoccupied molecular orbital (LUMO). The singlet excited state energy (E_S1_) was obtained from the intersection wavelength λ_c_ of the UV–Vis absorption and fluorescence emission spectra:[8]

$$\begin{aligned} E_{S1}\left( eV \right)=\frac{hc}{\lambda_{c}}\#\left( 10 \right) \end{aligned}$$

where h is Planck’s constant and c is the speed of light in vacuum. Using h ≈ 4.136 ×10^−15^ eV·s and c ≈ 3 × 10^8^ m/s, To express the energy per mole in kcal/mol, then we use the conversion 1 eV = 23.0605 kcal/mol, so:

$$\begin{aligned} E_{S1}\left( {kcal}/{mol} \right)\approx\frac{1240}{\lambda_{c}}\times23.0605\#\left( 11 \right) \end{aligned}$$

Singlet excited-state cleavage enthalpy (ΔH_cleavage S1_) and singlet excited-state cleavage enthalpy (ΔH_cleavage T1_) were calculated as:

$$\begin{aligned} {\Delta H}_{cleavage S1}=BDE-E_{S1}\#\left( 12 \right) \end{aligned}$$

$$\begin{aligned} {\Delta H}_{cleavage T1}=BDE-E_{T1}\#\left( 13 \right) \end{aligned}$$

For triplet–triplet energy transfer (TTET), the triplet energy offset was:

$$\begin{aligned} {\Delta E}_{T1}\left( {kcal}/{mol} \right)={\Delta E}_{T1}\left( Acceptor \right)-{\Delta E}_{T1}\left( Donor \right)\#\left（ 14 \right） \end{aligned}$$

### 1.12. Cell confluence assay

C_2_C_12_ myoblasts were seeded into 96-well plates at 1.0 × 10^4^ cells per well in complete growth medium (DMEM high glucose with 10 % fetal bovine serum and 1 % penicillin–streptomycin). After 4 h, the medium was replaced with fresh growth medium containing photoinitiator as indicated: ITX, BE2, EDB, ITX+BE2, or ITX+EDB. Photoinitiators were prepared as DMSO stock solutions and diluted into medium. Final assay concentrations were 25 or 50 mM per component. Cell proliferation was monitored for 40 h using an Incucyte SX1 live-cell imaging system with image acquisition every 2 h, and for each condition phase-contrast images were analyzed with the Incucyte software to determine the percentage of cell confluency over time.

### 1.13. Statistical analysis

Unless otherwise stated, quantitative experiments were performed in triplicate (n = 3). For real-time FTIR photopolymerization, conversion–time curves were obtained after baseline correction of the acrylate absorption band. The maximum polymerization rate (R_max_) was calculated from the steepest region of the conversion curve, and the final conversion was taken from the plateau region or from the value at the specified irradiation time. No data transformation or normalization was applied unless stated otherwise. Data were checked for obvious technical or instrumental errors, and no data points were excluded unless such errors were identified.

Data are presented as mean ± standard deviation (SD) or as box plots, as indicated in the figure legends. Statistical differences among multiple groups were analyzed by one-way analysis of variance (one-way ANOVA) followed by Tukey’s multiple-comparison test when appropriate. Tests were two-sided, and p < 0.05 was considered statistically significant. Significance levels are indicated as *p < 0.05, **p < 0.01 and ***p < 0.001. The suitability of parametric analysis was evaluated by checking data distribution and variance consistency before analysis. Statistical analysis and graph preparation were performed using Origin software.

## 2. Supplementary data

Figure S1. (a) Solubility of BEs (2 × 10^−5^ mol/g) in TMPTA, (b, c) photopolymerization performance of BEs/TMPTA formulations under LED@365 nm and LED@405 nm, respectively. (d) Solubility of BEs (2 × 10^−5^ mol/g) in ETPTA, and (e, f) Photopolymerization performance of BEs/ETPTA formulations under LED@365 nm and LED@405 nm, respectively.

Figure S2. Determination of excited singlet state energies for (a) BE1, (b) BE2 and (c) BE3. The excitation wavelengths (𝜆_ex_) for the fluorescence emission spectra of BE1, BE2, and BE3 are 279 nm, 307 nm, and 305 nm, respectively. All absorption and emission spectra were normalized to their maximum intensity for estimation of singlet-state energies.

Figure S3. Normalized spectral overlap between the fluorescence emission spectra of different photosensitizers, including (a) ITX, (b) BP, (c) CQ and (d) DBA, and the UV–Vis absorption spectrum of BE2. All absorption and emission spectra were normalized to their maximum intensity for comparison of spectral overlap. The excitation wavelengths (𝜆_ex_) for the fluorescence emission spectra of ITX, BP, CQ and DBA are 383 nm, 251 nm, 468 nm and 385 nm, respectively.

Figure S4. (a) Cyclic voltammogram of BE2 (vs. SCE), (b) Schematic illustration of the two possible electron transfer pathways between the photosensitizer (PS) and BE2, where PS* can act as either an electron donor or acceptor, and (c) HOMO and LUMO energy levels and orbital distributions of BEs, ITX, and BP calculated at the UB3LYP/6−31G* level (isovalue = 0.02 a.u.).

Table S1. The calculated ΔG_ET_ values.

| Pathway | Donor / Acceptor | ΔG_ET_ (eV) |
| --- | --- | --- |
| PS* → BE2 | ITX / BE2 | +0.34 |
| PS* → BE2 | BP / BE2 | +1.79 |
| BE2 → PS* | BE2 / ITX | +0.43 |
| BE2 → PS* | BE2 / BP | +0.64 |

Figure S5. (a) Fluorescence quenching of BE2+ITX and (b) Stern–Volmer treatment for fluorescence quenching.

Figure S6. Fluorescence decay curves for (a) BE1, (b) BE2 and (c) BE3.

Figure S7. Steady state photolysis of (a) BE1, (b) BE3, (c) BE1+ITX, and (d) BE3+ITX systems under LED@405 nm irradiation.

Figure S8. (a) Photopolymerization of thick samples of ETPTA containing different photoinitiating systems under sunlight illumination, (b) the weather temperature, (c) the ultraviolet index, and (d) the solar radiation on the day of the test (the experiment took place on April 10, 2025 from 12:00 to 14:00 at 15 Rue Jean Starcky, Mulhouse).

Table S2. Maximum polymerization rates and conversion of different initiation systems (2 × 10^−5^ mol/g BEs + 2 × 10^−5^ mol/g ITX).

|  | BE1+ITX | BE2+ITX | BE3+ITX | ITX |
| --- | --- | --- | --- | --- |
| $\frac{R_{p}}{[M_{0}]} \times100 (s^{-1})$ | 8.7 ± 1.2 | 13.4 ± 1.1 | 6.9 ± 0.5 | 4.3 ± 0.1 |
| Conversion (%) | 92.5 ± 0.4 | 94.6 ± 0.3 | 92.6 ± 0.5 | 90.7 ± 0.3 |

Figure S9. (a) Polymerization depth of ETPTA samples containing different photoinitiating systems under various light sources, (b) the weather temperature, (c) the ultraviolet index and (d) the solar radiation on the day of the test (the experiment took place on September 5, 2025 from 13:00 to 15:00 at 15 Rue Jean Starcky, Mulhouse).

Figure S10. (a) Photopolymerization of thick samples of ETPTA containing different photoinitiating systems under LED@405 nm illumination, and (b) photopolymerization kinetics of ETPTA thick samples containing different photoinitiating systems (2 × 10^–5^ mol/g 938/EDB/BE2 + 2 × 10^–5^ mol/g ITX) under LED@405 nm irradiation.

Figure S11. ESR spectra of different photoinitiating systems (a) BE2, (b) ITX, (c) CQ and (d) BE2+CQ under LED@405 nm irradiation. For direct comparison, the y-axis range were all kept identical to that used for the BE2+ITX ESR spectrum in Figure 4a.

Figure S12. FTIR spectra of (a, b) 2 × 10^−5^ mol/g BEs + 2 × 10^−5^ mol /g ITX and (c) 2 × 10^−5^ mol /g ITX initiation system of thin samples at 10 s and 60 s.

Figure S13. (a) Photopolymerization of the formulation for 3D printing using ETPTA as monomer (4 × 10^−6^ mol/g BE2 + 4 × 10^−6^ mol/g ITX), (b, c) letters printed via DLW with corresponding optical profilometry, and (d) Jacobs working curves with linear fits (for ETPTA containing 4 × 10^−6^ mol/g EDB + 4 × 10^−6^ mol/g ITX) of LCD printer.

Figure S14. (a, b) SEM images of the printed boat roof viewed from above and from the side, (c, d) SEM images of the printed castle roof, (e) 3D pixel‑art cat model used for DLP printing and (f) structures obtained, (g) castle structures printed LCD printer and (h, i) SEM image of the printed castle model viewed from side and from the above.

Figure S15. Real-time cell confluency of C_2_C_12_ myoblasts cultured for 40 h in growth medium containing (a) ITX, (b) BE2 or EDB at 25 or 50 mM, compared with untreated control cells.

## 3. General informations about synthetic BEs

### 3.1. (*E/Z*)-OX1

Furfural (2.0 g, 20.8 mmol, 1.0 eq.), hydroxylamine hydrochloride (2.0 g, 29.12 mmol, 1.4 eq.) and triethylamine (3.0 mL, 22.88 mmol, 1.1 eq.) were added separately to a dry reaction with magnetic stirrer, without additional solvent. The reaction was carried out in 55 °C for 12 h. After the reaction completed, the reaction was extracted in ethyl acetate. The organic layer was dried over MgSO_4_, filtered, concentrated in *vacuo* and the residue was purified by column chromatography (petroleum ether / ethyl acetate: 8/1) to afford the desired product **(*E/Z*)-OX1** (2.14 g, 92 %, *E/Z*: 1/1).

**(*E*)-OX1** ^1^H NMR (400 MHz, DMSO-d6): *δ* 11.78 (s, 1H), 7.77-7.75 (m, 1H), 7.52 (s, 1H), 7.20-7.19 (m, 1H), 6.63-6.61 (m, 1H).^13^C NMR (101 MHz, CDCl_3_): *δ* 145.2 (C), 143.5 (CHaro.), 137.1 (HCN), 118.3 (CHaro.), 112.4 (CHaro.).

**(*Z*)-OX1** ^1^H NMR (400 MHz, CDCl_3_): *δ* 11.23 (s, 1H), 8.01 (s, 1H), 7.75-7.74 (m, 1H), 6.70-6.69 (m, 1H) 6.56-6.55 (m, 1H). ^13^C NMR (101 MHz, CDCl_3_): *δ* 147.2 (C), 143.5 (CHaro.), 140.3 (HCN), 112.8 (CHaro.), 111.6 (CHaro.).

Analyses are in accordance with the literature.[9]

### 3.2. (*E/Z*)-BE1

To a stirred solution of **(*E/Z*)-OX1** (2.1 g, 19.1 mmol, 1.0 eq.) in triethylamine (3.5 mL, 28.8 mmol, 1.5 eq.) under argon at 0 °C, benzoyl chloride (2.2 mL, 19.2 mmol, 1.0 eq.) was added. The reaction was carried out in 40 °C during 2 h. After the reaction completed, the mixture was extracted with ethyl acetate and dried over MgSO_4_. The residue was purified by column chromatography (petroleum ether / ethyl acetate: 85/15) to afford the desired product **(*E/Z*)-BE1** (0.84 g, quant. *E/Z*: 1/1)

**(*E/Z*)-BE1** ^1^H NMR (400 MHz, CDCl_3_) *δ* 8.40 (s, 1H *E*), 8.25 – 8.05 (m, 4H, *Z/E*), 7.89 (s, 1H, *Z*), 7.76 – 7.42 (m, 8H Z/E), 7.30 (d, *J* = 3.6 Hz, 1H, *Z*), 6.97 (d, *J* = 3.5 Hz, 1H, *E*), 6.60 (dd, *J* = 3.7, 1.7 Hz, 1H, *Z*), 6.52 (dd, *J* = 3.5, 1.8 Hz, 1H, *E*). ^13^C NMR (101 MHz, CDCl_3_) δ 164.0 (C), 163.7 (C), 146.2 (CHaro.), 146.1 (CHaro.), 145.6 (CHaro.), 145.3 (C), 144.3 (C), 142.4 (CHaro.), 133.6 (CHaro.), 133.5 (CHaro.), 129.6 (CHaro.), 129.8 (CHaro.), 128.8 (CHaro.), 128.7 (C), 128.6 (CHaro.), 128.5 (C), 120.3 (CHaro.), 116.5 (CHaro.), 112.9 (CHaro.), 112.3 (CHaro.). HRMS (ESI): *m/z* calculated for C_12_H_9_NO_3_Na^+^: [M + Na]^+^; found: 238.0475.

**^1^H, ^13^C spectra and HRMS**

### 3.3. Vanillin OTBS

TBSCl (1.4 mL, 8.58 mmol, 1.3 eq.) was added to a solution of vanillin (1.0 g, 6.6 mmol, 1.0 eq.) in DCM (20 mL) with imidazole (1.12 g, 16.5 mmol, 2.5 eq.) and stirred at room temperature for 3 h. After the completion of the reaction, distilled water was added and organic was extracted with DCM. The extracted organic layers were dried over anhydrous MgSO_4_ and concentrated under reduced pressure. This concentrate was purified by column chromatography (petroleum ether / ethyl acetate: 8/2) to obtain the desired product (1.01 g, 58 %).

**Vanillin OTBS** ^1^H NMR (400 MHz, CDCl_3_): *δ* 9.85 (s, 1H), 7.38 (m, 2H), 6.96 (d, 1H), 3.87 (s, 3H), 1.00 (s, 9H), 0.19 (s, 6H). Analyses are in accordance with the literature.[10]

### 3.4. (*E*)-OX2

**Vanillin OTBS** (6.62 g, 26.4 mmol, 1.0 eq.), hydroxylamine hydrochloride (2.57 g, 36.96 mmol, 1.4 eq.) and triethylamine (3.5 mL, 29.04 mmol, 1.1 eq.) were added successively to a dry flask with magnetic stirrer, without additional solvent. The reaction was carried out in 55 °C for 12 h. After this time, the reaction was extracted with ethyl acetate. The organic layer was dried over MgSO_4_, filtered, concentrated in *vacuo* and the residue was purified by column chromatography (petroleum ether / ethyl acetate: 8/1) to afford the desired product **(*E*)-OX2** (6.68 g, q.).

**(*E*)-OX2** ^1^H NMR (400 MHz, CDCl_3_): *δ* 8.08 (s, 1H), 7.19-7.17 (m, 1H), 6.98-6.95 (m, 1H) 6.86-6.83 (m, 1H), 3.83 (s, 3H), 1.00 (s, 9H), 0.17 (s, 6H). ^13^C NMR (101 MHz, CDCl_3_): *δ* 151.5 (C), 150.5 (Cq-OCH_3_), 147.3 (HCN), 125.8 (C), 121.6 (CHaro.), 121.0 (CHaro.), 109.2 (CHaro.), 55.6 (O-CH_3_), 25.8 (3xCH_3_), 18.6 (C), -4.5 (2xSiCH_3_). HRMS (ESI): m/z calculated for C_14_H_23_NO_3_SiNa^+^: [M + Na]^+^; found: 304.1338.

**^1^H, ^13^C spectra and HRMS**

### 3.5. (*E*)-BE2-TBS

**(*E*)-OX2** (6.68 g, 26.9 mmol, 1.0 eq.) was dissolved in triethylamine (4.9 mL, 40.35 mmol, 1.5 eq.) under argon. Then at 0 °C, benzoyl chloride (3.1 mL, 26.9 mmol, 1.0 eq.) was added. The reaction was carried out in 40 °C during 2 h. After the reaction completed, the mixture was extracted with ethyl acetate and dried over MgSO_4_. The residue was purified by column chromatography (petroleum ether / ethyl acetate: 85/15) to afford the desired product **(*E*)-BE2-TBS** (2.96 g, 27 %).

**(*E*)-BE2-TBS** ^1^H NMR (400 MHz, CDCl_3_): *δ* 8.47 (s, 1H), 8.14-8.12 (m, 2H), 7.63-7.58 (m, 1H), 7.51-7.47 (m, 3H), 7.11-7.09 (m, 1H), 6.90-6.88 (m, 1H), 3.88 (s, 3H), 1.00 (s, 9H), 0.18 (s, 6H). ^13^C NMR (101 MHz, CDCl_3_): *δ* 164.3 (CO), 157.0 (C), 151.7 (HCN), 149.1 (C), 133.5 (CHaro.), 129.8 (2xCHaro.), 128.9 (C), 128.7 (2xCHaro.), 124.0 (C), 123.8 (CHaro.), 120.9 (CHaro.), 109.9 (CHaro.), 55.8 (O-CH_3_), 25.8 (3xCH_3_), 18.6 (C), -4.5 (2xCH_3_). HRMS (ESI): *m/z* calculated for C_21_H_27_NO_4_SiNa^+^: [M + Na]^+^; found: 408.1598.

**^1^H, ^13^C spectra and HRMS**

### 3.6. (*E*)-BE2

To a stirred solution of **(*E*)-BE2-TBS** (0.731 g, 1.9 mmol, 1.0 eq.) in THF (33 mL) under argon was added at 0 °C drop by drop, TBAF (3.8 mL, 3.8 mmol, 2.0 eq.). After 10 minutes, the reaction mixture was diluted with AcOEt and washed with water. The organic phase was dried over MgSO_4_. The residue was purified by column chromatography (petroleum ether / ethyl acetate: 5/5) to afford the desired product **(*E*)-BE2** (515 mg,q.).

**(*E*)-BE2** ^1^H NMR (400 MHz, CDCl_3_): *δ* 8.46 (s, 1H), 8.14-8.11 (m, 2H), 7.62-7.59 (m, 1H), 7.52-7.46 (m, 3H), 7.12-7.09 (m, 1H), 6.97-6.95 (m, 1H), 3.94 (s, 3H). ^13^C NMR (101 MHz, CDCl_3_): *δ* 164.4 (CO), 156.9 (HCN), 149.5 (C), 147.3 (C), 133.5 (CHaro.), 129.8 (2xCHaro.), 128.9 (C), 128.7 (2xCHaro.), 125.1 (C), 122.4 (CHaro.), 114.5 (CHaro.), 108.4 (CHaro.), 56.3 (CH_3_). HRMS (ESI): *m/z* calculated for C_15_H_13_NO_4_Na^+^: [M + Na]^+^; found: 294.0736.

**^1^H, ^13^C spectra and HRMS**

### 3.7. Syringaldehyde OTBS

To a solution of syringaldehyde (4.00 g, 22.0 mmol, 1.0 eq.) in DMF (80 mL) was added imidazole (2.99 g, 44.0 mmol, 2.0 eq.) and TBSCl (5.64 g, 37.4 mmol, 1.7 eq.). The mixture was stirred at room temperature for 30 min. The mixture was diluted with H_2_O and extracted with ethyl acetate three time. The organic extracts were combined, dried over MgSO_4_, filtered, concentrated in vacuo and the residue was then purified by column chromatography (petroleum ether / ethyl acetate: 8/2) to afford the desired product (6.31 g, 96 %).

**Syringaldehyde OTBS** ^1^H NMR (400 MHz, CDCl_3_): *δ* 9.82 (s, 1H), 7.10 (s, 2H), 3.87 (s, 6H), 1.01 (s, 9H), 0.16 (s, 6H). Analyses are in accordance with the literature^[1]^.

### 3.8. (*E*)-OX3

**Syringaldehyde OTBS** (4.00 g, 13.5 mmol, 1.0 eq.), hydroxylamine hydrochloride (1.31 g, 18.9 mmol, 1.4 eq.) and triethylamine (1.8 mL, 14.9 mmol, 1.1 eq.) were added successively to a dry flask with magnetic stirrer, without additional solvent. The reaction was carried out in 55 °C for 12 h. After the reaction completed, the reaction was extracted with ethyl acetate. The organic layer was dried over MgSO_4_, filtered, concentrated in *vacuo* and the residue was purified by column chromatography (petroleum ether / ethyl acetate: 85/15) to afford the desired product **(*E*)-OX3** (1.33 g, 33 %).

**(*E*)-OX3** ^1^H NMR (400 MHz, CDCl_3_): *δ* 8.05 (s, 1H), 6.78 (s, 2H), 3.81 (s, 6H), 1.01, (s, 9H), 0.14 (s, 6H). ^13^C NMR (101 MHz, CDCl_3_): *δ* 152.0 (2xC), 150.7 (NCH), 136.6 (C), 124.6 (C), 104.4 (2xCHaro.), 55.9 (2xO-CH_3_), 25.9 (3xCH_3_), 18.9 (C), -4.5 (2xSi-CH_3_). HRMS (ESI): *m/z* calculated for C_15_H_25_NO_4_SiNa^+^: [M + Na]^+^; found: 334.1443.

**^1^H, ^13^C spectra and HRMS**

### 3.9. (*E*)-BE3-TBS

**(*E*)-OX3** (1.33 g, 4.5 mmol, 1.0 eq.) was dissolved in triethylamine (0.84 mL, 6.75 mmol, 1.5 eq.) under argon. Then at 0 °C, benzoyl chloride was added. The reaction was carried out in 40 °C during 2 h. After the reaction completed, the reaction mixture was extracted with ethyl acetate and dried over MgSO_4_. The residue was purified by column chromatography (petroleum ether / ethyl acetate: 85/15) to afford the desired product **(*E*)-BE3-TBS** (1.66 g, 89 %).

**(*E*)-BE3-TBS** ^1^H NMR (400 MHz, CDCl_3_): *δ* 8.45 (s, 1H), 8.14-8.11 (m, 2H), 7.63-7.59 (m, 1H), 7.52-7.49 (m, 2H), 7.00 (s, 2H), 3.85 (s, 6H), 1.02 (s, 9H), 0.16 (s, 6H). ^13^C NMR (101 MHz, CDCl_3_): *δ* 164.3 (CO), 157.3 (2xC), 152.1 (NCH), 138.3 (C), 133.5 (CHaro.), 129.8 (2xCHaro.), 129.0 (C), 128.7 (2xCHaro.), 122.5 (C), 105.9 (2xCHaro.), 56.1 (2xOCH_3_), 25.9 (3xCH_3_), 18.90 (C), -4.5 (2xSiCH_3_). HRMS (ESI): *m/z* calculated for C_22_H_29_NO_5_SiNa^+^: [M + Na]^+^; found: 438.1707.

**^1^H, ^13^C spectra and HRMS**

### 3.10. (*E*)-BE3

To a stirred solution of **(*E*)-BE3-TBS** (0.341 g, 0.8 mmol, 1.0 eq.) in THF (14 mL) was added at 0 °C, TBAF drop by drop (1.6 mL, 1.6 mmol, 2.0 eq.). After 10 minutes, the reaction mixture was diluted in AcOEt and washed with water. The reaction mixture was dried over MgSO_4_. The residue was purified by column chromatography (petroleum ether / ethyl acetate: 5/5) to afford the desired product **(*E*)-BE3** (202 mg, 88 %).

**(*E*)-BE3** ^1^H NMR (400 MHz, CDCl_3_): *δ* 8.44 (s, 1H), 8.13-8.10 (m, 2H), 7.62-7.58 (m, 1H), 7.50-7.46 (m, 2H), 7.04 (s, 2H), 3.94 (s, 6 H). ^13^C NMR (101 MHz, CDCl_3_): 164.3 (CO), 157.1 (NCH), 147.4 (2xC), 138.5 (C), 135.5 (CHaro.), 129.8 (2xCHaro.), 128.8 (C), 128.7 (2xCHaro.), 121.3 (C), 105.7 (2x CHaro.), 55.7 (2xCH_3_). HRMS (ESI): *m/z* calculated for C_16_H_15_NO_5_Na^+^: [M + Na]^+^; found: 324.0842.

**^1^H, ^13^C spectra and HRMS**

## 4. References

[1] O. Smirnova, S. Hwang, R. Sajzew, L. Ge, A. Reupert, V. Nozari, S. Savani, C. Chmelik, M.R. Reithofer, L. Wondraczek, J. Kärger, A. Knebel, Precise control over gas-transporting channels in zeolitic imidazolate framework glasses, Nature Materials 23 (2024) 262-270. https://doi.org/10.1038/s41563-023-01738-3

[2] Y. Zhang, B. Song, Z. Liu, A. Noon, C. Dietlin, F. Morlet-Savary, M. Schmitt, D. Gigmes, F. Dumur, J. Lalevée, High photoinitiating efficiency of benzothioxanthene-based oxime esters in photopolymerization via photocleavage and/or single electron transfer under visible light and sunlight, Angewandte Chemie International Edition 63 (2024) e202405337. https://doi.org/10.1002/anie.202405337

[3] P. Xiao, F. Dumur, B. Graff, D. Gigmes, J.P. Fouassier, J. Lalevée, Blue light sensitive dyes for various photopolymerization reactions: naphthalimide and naphthalic anhydride derivatives, Macromolecules 47 (2014) 601-608. https://doi.org/10.1021/ma402376x

[4] L. Niederst, X. Allonas, M. Morone, S. van den Branden, Employing Singlet-Singlet Energy Transfer for Boosting the Reactivity of Type I Photoinitiators in Radical Photopolymerization, Angewandte Chemie International Edition 63 (2024) e202412625. https://doi.org/10.1002/anie.202412625

[5] N. Liaros, S.A. Gutierrez Razo, M.D. Thum, H.M. Ogden, A.N. Zeppuhar, S. Wolf, T. Baldacchini, M.J. Kelley, J.S. Petersen, D.E. Falvey, A.S. Mullin, J.T. Fourkas, Elucidating complex triplet-state dynamics in the model system isopropylthioxanthone, iScience 25 (2022). https://doi.org/10.1016/j.isci.2021.103600

[6] S. Islam, M. Sangermano, T.A. Klar, STED-Inspired Cationic Photoinhibition Lithography, The Journal of Physical Chemistry C 127 (2023) 18736-18744. https://doi.org/10.1021/acs.jpcc.3c04394

[7] T.J. Kolibaba, J.P. Killgore, B.W. Caplins, C.I. Higgins, U. Arp, C.C. Miller, D.L. Poster, Y. Zong, S. Broce, T. Wang, V. Talačka, J. Andersson, A. Davenport, M.A. Panzer, J.R. Tumbleston, J.M. Gonzalez, J. Huffstetler, B.R. Lund, K. Billerbeck, A.M. Clay, M.R. Fratarcangeli, H.J. Qi, D.H. Porcincula, L.B. Bezek, K. Kikuta, M.N. Pearlson, D.A. Walker, C.J. Long, E. Hasa, A. Aguirre-Soto, A. Celis-Guzman, D.E. Backman, R.L. Sridhar, K.A. Cavicchi, R.J. Viereckl, E. Tong, C.J. Hansen, D.M. Shah, C. Kinane, A. Pena-Francesch, C. Antonini, R. Chaudhary, G. Muraca, Y. Bensouda, Y. Zhang, X. Zhao, Results of an interlaboratory study on the working curve in vat photopolymerization, Additive Manufacturing 84 (2024) 104082. https://doi.org/10.1016/j.addma.2024.104082

[8] T. Torres, A. Gouloumis, D. Sanchez-Garcia, J. Jayawickramarajah, W. Seitz, D.M. Guldi, J.L. Sessler, Photophysical characterization of a cytidine–guanosine tethered phthalocyanine–fullerene dyad, Chemical Communications 3 (2007) 292-294. https://doi.org/10.1039/B613086K

[9] X. Jiang, X. Xu, Y. Lin, Y. Yan, P. Li, R. Bai, Y. Xie, A mild system for synthesis of aldoximes and ketoximes in the presence of N-hydroxyphthalimide in aqueous system, Tetrahedron 74 (2018) 5879-5885. https://doi.org/10.1016/j.tet.2018.08.013

[10] Y. Son, J. Kim, Y. Kim, S.-G. Chi, T. Kim, J. Yu, Discovery of dioxo-benzo[b]thiophene derivatives as potent YAP-TEAD interaction inhibitors for treating breast cancer, Bioorganic Chemistry 131 (2023) 106274. https://doi.org/10.1016/j.bioorg.2022.106274

1. Hu Gaojie, Liu Kai and J. Williams Lawrence, The Brosimum Allene: A Structural Revision. *Org. Lett.* **2008**, *10(23)*, 5493–5496
